# Supplementary material for: Lived experiences of caregivers of persons with epilepsy attending an epilepsy clinic at a tertiary hospital, eastern Uganda: A phenomenological approach
Source: PLoS One. 2023 Jul 18;18(7):e0274373. doi: 10.1371/journal.pone.0274373 (PMC10353802; doi:10.1371/journal.pone.0274373)
Supplement: S1 Data — (ZIP) [file pone.0274373.s001.zip › SOCIAL BURDEN.pdf]

Ca 8 1

SOCIAL BURDEN

Interviewer	I know that persons with epilepsy can get attacks (epileptic) anywhere at any time, how do these attacks influence your public relationship?
Respondent	*I fear when she fits , I don't feel comfortable being in public where there are many people*
Interviewer	Ok…. At times these epileptic attacks come in an untimely manner and when you are not prepared for it. ~~How do you feel about the unpredictable epileptic attacks?~~
Respondent	*I always think that the attack can come about anytime…….it makes me worry.*
Interviewer	How do you go about stigma while caring for a person with epilepsy?
Respondent	*I am strong because health workers tell me that the child will be fine.*
Interviewer	*What do you think you can do as a result of the challenges you face while caring for a person with epilepsy?* I bring her for treatment and pray to God
Respondent	*[Interruption child shouts] getting her treated and praying to God.*
Interviewer	*What can you do to a situation that calls for your absence from a person with epilepsy?*
Respondent	*I leave her under the aunts care she is the only one who can manage her, she feeds her, but other people don't like her, the aunt takes good care of her even if I am not around*
Interviewer	*Sometimes families get affected while caring for a person with epilepsy, How is your family affected?*
Respondent	*For us we feel we don't have any problem as a family.*

Case 2

SOCIAL BURDEN

Interviewer	Persons with epilepsy can get attacks (epileptic) anywhere at any time, how do these attacks influence your public relationship?
Respondent	*They don't come closer to her me I stay with her when she falls I keep her until she gets up and we walk back home.*
Interviewer	Ok…. At times these epileptic attacks come when you are not prepared for it. How do you feel about the unpredictable epileptic attacks?
Respondent	*I normally worry that she may fall in dangerous places, when the attack is to come she understands she can tell you today I don't feel fine whenever she tells me that a day can't pass without her getting an attack so I closely monitor her .*
Interviewer	How do you go about stigma while caring for a person with epilepsy?
Respondent	*I have to endure because it's my problem.*
Interviewer	*What do you think you can do as a result of the challenges you face while caring for a person with epilepsy?*
Respondent	*I ensure that she takes her drugs on time every day, I feed her on time because the drugs may treat her badly ,I also take her to different churches for prayers so that God may heal her.*
Interviewer	*What can you do to a situation that calls for your absence from a person with epilepsy?*
Respondent	*Now I know the time that she falls, when that time reaches I don't go anywhere I first keep her for 20 or 30 minutes before I go. When I go somewhere sometimes we go together but if I leave her behind I tell my elder son to take care of her well and I make sure that I come back soon.*
Interviewer	*Sometimes families get affected while caring for a person with epilepsy, How is your family affected?*
Respondent	*Sometimes my husband says he doesn't want her to be together with his children, he tells me to send her off so that she may go back to their home and I tell him that I can't let her go because her s step mother can't take care of her.*

Case 3

SOCIAL BURDEN

Interviewer	Persons with epilepsy can get attacks (epileptic) anywhere at any time, how do these attacks influence your public relationship?
Respondent	*I don't feel good I think more about the future I think about that ka....thing.*
Interviewer	At times these epileptic attacks come abruptly. How do you feel about the unpredictable epileptic attacks?
Respondent	*Really I don't feel well; if God allows my daughter to walk I may not leave her behind if I am cooking.*
Interviewer	How do you go about stigma while caring for a person with epilepsy?
Respondent	*Sometimes when I am going to church I carry her so that she doesn't go where there are people because I know my daughters condition.*
Interviewer	*What do you think you can do as a result of the challenges you face while caring for a person with epilepsy?*
Respondent	*I make sure that she doesn't disturb the aunties, uncles and other people who don't want to be touched Ahaha..........*
Interviewer	*What can you do to a situation that calls for your absence from a person with epilepsy?*
Respondent	*I have a sister together with my mother who can help in my absence.*
Interviewer	*Sometimes families get affected while caring for a person with epilepsy, How is your family affected?*
Respondent	*My family members take it easy and my husband helps me carry her when I am doing house hold chores.*

Case 4

SOCIAL BURDEN

Interviewer	Persons with epilepsy can get attacks (epileptic) anywhere at any time, how do these attacks influence your public relationship?
Respondent	*People don't treat me badly I relate well with them they don't isolate me.*
Interviewer	At times these epileptic attacks come abruptly. How do you feel about the unpredictable epileptic attacks?
Respondent	*I don't fear because these days he doesn't fit for the whole day like he used to do ,he can fit like for 3 minutes then he gets up If he also disturbs me a lot it's a sign that he is about to get an attack..*
Interviewer	Your son being a child I know sometimes he goes to play with fellow children in the neighbourhood.How do you go about stigma while caring for him?
Respondent	*Ok…………some people chase him and tell him to go back home, some say that he will infect their children I make sure that I restrict his movement ,he stays at home , he eats ,bathes, plays and sleeps thereafter.*
Interviewer	*What do you think you can do as a result of the challenges you face while caring for a person with epilepsy?*
Respondent	*I bring him for treatment on appointment days and I pray to God.*
Interviewer	*What can you do to a situation that calls for your absence from a person with epilepsy?*
Respondent	*I leave him with my daughter in law she is good and she takes good care of him just like me.*
Interviewer	*A god number of families get affected while caring for a person with epilepsy, How is your family affected?*
Respondent	*My family members are not bad the siblings feed him, they bathe and they don't shout at him .[Interruption phone ringing].*

Case 5

SOCIAL BURDEN

Interviewer	Persons with epilepsy can get attacks (epileptic) at any time in any place, how do these attacks influence your public relationship?
Respondent	*There are people who automatically understand the nature of this disease they come close to you and help you but there are those who fear and believe that when you go near those people who fall that thing will get hold of you also so they run away.*
Interviewer	At times these epileptic attacks come abruptly. How do you feel about the unpredictable epileptic attacks?
Respondent	Me I just carry him inside then I make him lie in a safe place I don't fear but those days I used to think that he wants to die.
Interviewer	Your grandson being a child I know sometimes he goes to play with fellow children in the neighborhood but he may be feared or chased to come back home. How do you go about stigma while caring for him?
Respondent	*Those words of theirs I just put them apart, for us we said no we have never divided things for the patient we share with him if it's eating we sit together and eat.*
Interviewer	*What do you think you can do as a result of the challenges you face while caring for a person with epilepsy?*
Respondent	*I restrict him from moving to people's homes since he likes fighting and eating you know children he can enter in someone's house and get something to eat, I also pray for him and bring him for treatment.*
Interviewer	*What can you do to a situation that calls for your absence from a person with epilepsy?*
Respondent	*When I go somewhere I have to come back home quickly I don't delay because I know these attacks come at any time.*
Interviewer	*A god number of families get affected while caring for a person with epilepsy, How is your family affected?*
Respondent	*My family members fear him especially when he gets an attack his attacks are scaring they keep on telling me to take the boy back to his mother but I tell them I can't take the boy back because his mother is helpless.*

Case 6

SOCIAL BURDEN

Interviewer	Persons with epilepsy can get attacks (epileptic) at any time in any place, how do these attacks influence your public relationship?
Respondent	*Okay……… it has ever happened but people assisted me. Sometimes it happens when he goes to sit with his friends but they always carry him and bring him back home*
Interviewer	At times these epileptic attacks come abruptly. How do you feel about the unpredictable epileptic attacks?
Respondent	I used to feel discouraged that he would die those days but now am used I find it normal and I don't fear.
Interviewer	How do you go about stigma while caring for your husband?
Respondent	*The doctor told me this disease is not infectious we share the same cups and plates with him and I wash his bed sheets when he urinates*
Interviewer	*What do you think you can do as a result of the challenges you face while caring for a person with epilepsy?*
Respondent	*If I have money, I can do a small business to assist my family. Some times when he cannot bathe I bathe him.*
Interviewer	*What can you do to a situation that calls for your absence from a person with epilepsy?*
Respondent	*When he is okay I prepare some meal and leave for him but when he is not fine, I tell my mother in law to help me and look after him when I am away*
Interviewer	*Families get affected while caring for a person with epilepsy, How is your family affected?*
Respondent	*My children ask me mummy our daddy is sick will he get well or he will die. Some of his relatives pay us a visit when they hear that he is sick.*

Case 7

SOCIAL BURDEN

Interviewer	Persons with epilepsy can get attacks (epileptic) at any time in any place, how do these attacks influence your public relationship?
Respondent	*That is so shameful I feel sorry because if she is in a public place and she makes noise she begins fidgeting then she falls down upon recovering she gets up with a lot of energy like a mad woman and urinates on herself many people laugh and you just have to be strong.*
Interviewer	At times these epileptic attacks come abruptly. How do you feel about the unpredictable epileptic attacks?
Respondent	Me I wonder I don't have any day that she sleeps without getting an attack she fits every night that thing took away my peace but the good thing I resorted to praying this is not an easy thing if it occurs in a public place like in town you just say God its better you take her because I have nothing to do.
Interviewer	How do you go about stigma while caring for your daughter ?
Respondent	*like me they defeated me I don't have any thing that I do [interruption phone ringing]*
Interviewer	*What do you think you can do as a result of the challenges you face while caring for a person with epilepsy?*
Respondent	*Praying and taking her for treatment has also strengthened me*
Interviewer	*What can you do to a situation that calls for your absence from a person with epilepsy?*
Respondent	*I live her with my sister in case I go home and it so happens that I am caught up with rain or darkness I may decide to spend a night but I worry a lot. It's not easy you can't move freely or do a business*
Interviewer	*A good number of families get affected while caring for a person with epilepsy, How is your family affected?*
Respondent	*Men leave me because you can be there with your loved one and want to have some good time together but that night the child's condition worsens my daughter's illness has made me fail to settle in marriage all the men I get leave me once they realize that she has that disease.*

Case 8

SOCIAL BURDEN

Interviewer	Persons with epilepsy can get attacks (epileptic) at any time in any place, how do these attacks influence your public relationship?
Respondent	*If she falls in a public place some people run away, but those who know about these disease come and help me.*
Interviewer	At times these epileptic attacks come abruptly. How do you feel about the unpredictable epileptic attacks?
Respondent	All the time I am worried because the attack does not alert you and I am scared one day she may fall in the dangerous place.
Interviewer	How do you go about stigma while caring for your daughter ?
Respondent	*When I see the disease is progressing I take her to the hospital where they can do some checkups that strengthens me because they tell me what they are treating her for. I also pray because prayers strengthen me and enable me to endure with the patient's condition because when you go to the witch doctors they can take your money and the patients remains suffering.so I pray as I take her for treatment.*
Interviewer	*What do you think you can do as a result of the challenges you face while caring for a person with epilepsy?*
Respondent	*You worry all the time even when you cook food you eat but you don`t get satisfied. I seek medical health and I go for prayers as well.*
Interviewer	*What can you do to a situation that calls for your absence from a person with epilepsy?*
Respondent	*When I get a problem I first take care of her, I prepare for her food then I go away. When a problem requires me to spend a night away from home I excuse myself and tell them I have a patient at home, and there is no one to help her, so I get back home quickly and take care of her.*
Interviewer	*Families get affected while caring for a person with epilepsy, How is your family affected?*
Respondent	*My children cannot isolate themselves from their sister, they show her love so that she is not affected because they know that she is sick.my husband worries because we were wishing her many good things as parents but now she can`t I achieve them.*

Case 9

SOCIAL BURDEN

Interviewer	Persons with epilepsy can get attacks (epileptic) at any time in any place, how do these attacks influence your public relationship?
Respondent	*I feel bad, some people ran and went away this happened when were at church then some people felt pity they helped me and told me to take her for treatment when she became stable.*
Interviewer	At times these epileptic attacks come abruptly. How do you feel about the unpredictable epileptic attacks?
Respondent	I feel bad I pray to God that it may end.
Interviewer	How do you go about stigma while caring for your daughter ?
Respondent	*Me I pray, I only pray because there is nothing else that I can do.*
Interviewer	*What do you think you can do as a result of the challenges you face while caring for a person with epilepsy?*
Respondent	*I resort to prayers.*
Interviewer	*What can you do to a situation that calls for your absence from a person with epilepsy?*
Respondent	*When I go somewhere I normally leave her with her sister.*
Interviewer	*Families get affected while caring for a person with epilepsy, How is your family affected?*
Respondent	*They feel bad, they want her to get well but they stay and play with her in fact they love her so much.*

Case 10

SOCIAL BURDEN

Interviewer	Persons with epilepsy can get attacks (epileptic) at any time in any place, how do these attacks influence your public relationship?
Respondent	*Some people fear, but some help those who fear run away because they think that they can get infected. Other people help me carry him when he gets an attack or they give me money to bring him to the hospital.*
Interviewer	At times these epileptic attacks come abruptly. How do you feel about the unpredictable epileptic attacks?
Respondent	I feel bad, I fear I try to be near all the time so that I can help in case he gets attacked.
Interviewer	How do you go about stigma while caring for your son ?
Respondent	*[interruption people talking from outside] I pray to God to help me and make strong and I also go to people whom I know like health workers, I talk to them and they encourage me.*
Interviewer	*What do you think you can do as a result of the challenges you face while caring for a person with epilepsy?*
Respondent	*I resort to prayers, I get help from health workers.*
Interviewer	*What can you do to a situation that calls for your absence from a person with epilepsy?*
Respondent	*When I go somewhere I normally leave him with my wife or my brother.*
Interviewer	*Families get affected while caring for a person with epilepsy, How is your family affected?*
Respondent	*They feel bad but they are strong because there is no one else who can help him like his family.*

Case 11

SOCIAL BURDEN

Interviewer	Persons with epilepsy can get attacks (epileptic) at any time in any place, how do these attacks influence your public relationship?
Respondent	*It isolates you it segregates you they say that this one aha!.... you look at the patient she is badly off they really categorize you to be suffering from the same condition and you don`t feel like being with them someone can even say that you will give that thing to us. People don`t want to be with you.*
Interviewer	At times these epileptic attacks come abruptly. How do you feel about the unpredictable epileptic attacks?
Respondent	You don`t feel okey because your not prepared for it, you feel pain because if there was some sign going to show that your client is going to get an attack you would first organize how to sit down or lie down because she can be injured and from nowhere people will start criticizing you also why didn`t you prepare as if you knew, they say how can you allow some body to get this.those who are talking they don`t know how this condition comes about.
Interviewer	How do you go about stigma while caring for your person ?
Respondent	*By counseling them, by telling them this thing is not contagious and it does not come by choice I tell them they need to help someone in an attack so there you find them saying it is ok.*
Interviewer	*What do you think you can do as a result of the challenges you face while caring for a person with epilepsy?*
Respondent	*I would lobby for resources to open peoples mind by health educating, counseling them because when someone see`s this continuous sensitization of the community, there is need to get more care givers because you can`t be occupied the whole day even if you are in your home you are called then they say hallo you come and see your patient.*
Interviewer	*What can you do to a situation that calls for your absence from a person with epilepsy?*
Respondent	*I tell people who are around what to do but they tell me don`t take long there because for us we fear this patient. So that means even if you go away your not totally free.*
Interviewer	*Families get affected while caring for a person with epilepsy, How is your family affected?*
Respondent	*The families affected because financially, socially you are occupied you can move for other things even at night you can be called to attend to the patient then socially may be you have just gone home again they call you it does not allow you to be at home.*

Case 12

SOCIAL BURDEN

Interviewer	Persons with epilepsy can get attacks (epileptic) at any time in any place, how do these attacks influence your public relationship?
Respondent	*They ask me if that is how he is, most people fear to touch him others feel sorry but I touch him and take care of my son alone.*
Interviewer	At times these epileptic attacks come abruptly. How do you feel about the unpredictable epileptic attacks?
Respondent	I don`t feel happy about it but I got used to the situation, I don`t get annoyed of the unpredictable epileptic attacks because I know they come anywhere at any time.
Interviewer	How do you go about stigma while caring for your person ?
Respondent	*In our church we have our pastor who teaches about search things when someone gets a problem and you segregate that person or say I don`t like that you be telling the devil to attack you, this has helped people to understand that the sickness is not infectious and relate well with him except they don`t touch my child.*
Interviewer	*What do you think you can do as a result of the challenges you face while caring for a person with epilepsy?*
Respondent	*Like me I say if God wills he may heal him or take him, I am free in my heart I pray and I have left God to take care of him.*
Interviewer	*What can you do to a situation that calls for your absence from a person with epilepsy?*
Respondent	*I leave him under the father's care and elder siblings if I am away the Dad takes care of him, he loves him more than me.*
Interviewer	*Families get affected while caring for a person with epilepsy, How is your family affected?*
Respondent	*They are all happy the Dady and his siblings are very supportive and show him love.*

Case 13

SOCIAL BURDEN

Interviewer	Persons with epilepsy can get attacks (epileptic) at any time in any place, how do these attacks influence your public relationship?
Respondent	*Some people have knowledge about the disease whereas other people don't know; those who know hold him and help me when the attack comes whereas those who don't know run away.*
Interviewer	At times these epileptic attacks come abruptly. How do you feel about the unpredictable epileptic attacks?
Respondent	It treats me badly i feel and wish that it stops right now I do not fear I have handed him over to God when he gets attacked I pray.
Interviewer	How do you go about stigma while caring for your person ?
Respondent	*Some people say that he is Mad those ones I leave them to God because they don't know what they speak may God forgive them.*
Interviewer	*What do you think you can do as a result of the challenges you face while caring for a person with epilepsy?*
Respondent	*I can pray and also administer treatment to the baby.*
Interviewer	*What can you do to a situation that calls for your absence from a person with epilepsy?*
Respondent	*If I am going somewhere I normally go with the sick child and I leave the younger one behind with the siblings because he does not disturb them.*
Interviewer	*Families get affected while caring for a person with epilepsy, How is your family affected?*
Respondent	*The father prays and cries while wishing that his son gets well. His siblings want him to talk so that they can play and communicate well.*

Case 14

SOCIAL BURDEN

Interviewer	Persons with epilepsy can get attacks (epileptic) at any time in any place, how do these attacks influence your public relationship?
Respondent	*She studied up to primary four some children used to laugh at her and say that those are demos, that made her feel out of place since I don't want her to end up getting another problem, we decide to keep her home and we have bought charts and books that help children learn how to read and write for her so that she can also fit in the home and feel comfortable when people speak English.*
Interviewer	At times these epileptic attacks come abruptly. How do you feel about the unpredictable epileptic attacks?
Respondent	Obviously those ones scare me utmost, it mainly scares me when I struggle to do what I a m supposed to do and after doing it ,you see the thing coming at times I also feel stressed because I ask myself if by now I am around and I am here what if she is alone in the room and it so happens that it comes then what could have happened .
Interviewer	How do you go about stigma while caring for your person ?
Respondent	*The first thing I had to do was to tell her that she is not the first one, many people are like her and others are even worse so if such things happen just look at them and ignore just know that God loves you, if it wasn't the case then you would not be alive because many people are attacked and they just die once then God loves you even if they say that those are demons. We first gave her an opportunity to say whether she was comfortable staying in our home and she agreed before we brought her home.*
Interviewer	*What do you think you can do as a result of the challenges you face while caring for a person with epilepsy?*
Respondent	*Like me now as an individual nothing much ,but I can at least try to inquire .like today I inquired from the concerned people such that they can advise me on how to go about it.*
Interviewer	*What can you do to a situation that calls for your absence from a person with epilepsy?*
Respondent	*I have someone to help if I am not there this is someone who can take care of her but also when I am not around home I keep checking on her from time to time.*
Interviewer	*Families get affected while caring for a person with epilepsy, How is your family affected?*
Respondent	*Everyone is okay with her we don't take her as a stranger she is part of us.*

case 15

SOCIAL BURDEN

Interviewer	Persons with epilepsy can get attacks (epileptic) at any time in any place, how do these attacks influence your public relationship?
Respondent	*Many people wondered they had never seen an epileptic attack the day they witnessed him get an attack they asked me you mean that mzei is sick when it occurs in a public place you become discriminated.*
Interviewer	At times these epileptic attacks come abruptly. How do you feel about the unpredictable epileptic attacks?
Respondent	I feel like my heart is breaking, these attacks at times come at night when he is asleep and they are more severe my mum used to cry when she was taking care of him she used to call me more often and tell me my daughter help me and take your brother until we decided to relieve of the burden since she is aging.
Interviewer	How do you go about stigma while caring for your person ?
Respondent	*This way where we are we don't have many people, its only me, my husband and my children but they are at school they just come home for their holidays unlike in our home where mum has a large family in fact she used to face a hard time because my brother got used to having his own things even this way I tried to change his things and he asked for them .*
Interviewer	*What do you think you can do as a result of the challenges you face while caring for a person with epilepsy?*
Respondent	*I first leave him until he makes up his mind to do something and if he makes up his mind ,he does everything so perfectly even if you force him if he hasn`t made the decision he can`t do it .i came to learn that myself it takes him time to make up his mind himself if you give him time he can decide himself to wash the utensils, to clean the compound but you can`t tell him by force that you do this, when it`s too much I advise.*
Interviewer	*What can you do to a situation that calls for your absence from a person with epilepsy?*
Respondent	*That thing troubles me a lot ,I can`t be settled when I am away from him Ehe!,but when my husband is around we normally at list when he is not around then I should be there and if he is not around I am there we don`t stay with house helps may be they go just because of him as a fact it`s an a shaming disease someone experiences that they go away.*
Interviewer	*Families get affected while caring for a person with epilepsy, How is your family affected?*
Respondent	*My family doesn`t get affected in any way, we all love him and we stay well.*

case 16

SOCIAL BURDEN

Interviewer	Persons with epilepsy can get attacks (epileptic) at any time in any place, how do these attacks influence your public relationship?
Respondent	*He has ever been attacked they were going for a burial and it happened the people were helpful they helped to handle him then when he recovered there is somebody who knew him who had to escort him back home and mother proceeded for the burial.*
Interviewer	At times these epileptic attacks come abruptly. How do you feel about the unpredictable epileptic attacks?
Respondent	The worry is always if it attacks him in dangerous areas being an older person than us sometimes he cannot take the advice like you can tell him don`t ride a bicycle and there situations he insists and rides so there is that worry in case it happens on the road and you know how bodabodas ride and there are careless drivers.
Interviewer	How do you go about stigma while caring for your person?
Respondent	*For us we have got knowledge we have been sensitized actually me and my mum we get answers to all those questions and now that we know personally I endevour to sensitize the rest even those who think even if Mzee is on this treatment there those who say he is bewitched I talk to them that this thing is not through witchcraft.*
Interviewer	*What do you think you can do as a result of the challenges you face while caring for a person with epilepsy?*
Respondent	*May be if there is a way I keep sensitizing him and may be from medical, people when we come here they keep telling him about the does and don`ts, dangers if you did this and this may be as they tell him that he may change actually there is a scenario recent some are, there was a neighbor who had a son with the same he went to a dam to fetch water and he got an attack there he was lucky, people were passing there he witnessed it and these are some of the things we have been telling him don`t ride, so when he witnessed that he is a bit changing because initially used to go fishing and it is one of the things I am realizing one time I talked to him then he says even if it happens at my age I am old I am married I think he is likely to change.*
Interviewer	*What can you do to a situation that calls for your absence from a person with epilepsy?*
Respondent	*By sensitizing those who do not know about the disease.*
Interviewer	*Families get affected while caring for a person with epilepsy, How is your family affected?*
Respondent	*Since he has been kind of a bread winner so eheee!...there is a bit of struggling financially because we don`t want him to do garden work, he has been taking care of his animals and now we are discouraging him yet actually you find something else to do this is somebody who has been used to getting money we are telling him to sell animals and buy basic needs.*

Case 17

SOCIAL BURDEN

Interviewer	Persons with epilepsy can get attacks (epileptic) at any time in any place, how do these attacks influence your public relationship?
Respondent	*Sometimes I leave him at home but he goes to a trading centre when he gets an attack there he is helped by people who know him and me they hold him and put him down well when picks-up they call me to come for him they are used to his condition and they don`t run away from him.*
Interviewer	At times these epileptic attacks come abruptly. How do you feel about the unpredictable epileptic attacks?
Respondent	Like me I don`t fear him I hold him and place him down then I leave him until he gets well, I feel bad because I don`t know what time or which day the attack will come.
Interviewer	How do you go about stigma while caring for your person?
Respondent	*It affects me sometimes people run away some people talk about him, though I don`t hear them talk about him other people tell me but I trust God who brought this disease I don`t have what to do i leave him to God.*
Interviewer	*What do you think you can do as a result of the challenges you face while caring for a person with epilepsy?*
Respondent	*I pray.*
Interviewer	*What can you do to a situation that calls for your absence from a person with epilepsy?*
Respondent	*I leave him with the mother or his elder siblings.*
Interviewer	*Families get affected while caring for a person with epilepsy, How is your family affected?*
Respondent	*They are also affected they feel bad when their brother gets an attack, the attack can come when they are eating there all of them stop eating and they sleep hungry because they feel bad and their appetite is lost.*

Case 18

SOCIAL BURDEN

Interviewer	Persons with epilepsy can get attacks (epileptic) at any time in any place, how do these attacks influence your public relationship?
Respondent	*Usually she does not fall down I only saw her fall down once at home but then I cannot go around telling people who will go around laughing and my daughter will get a shamed I only tell a person who can help me, how can give me an idea of what to do, I cannot talk any how[mother laughing]*
Interviewer	At times these epileptic attacks come abruptly. How do you feel about the unpredictable epileptic attacks?
Respondent	I normally get many thoughts and wonder if that thing happens to get her when she is cooking she can fall on fire and get burnt or she can fall down and fail to breath well the fact that she is staying in the hostel alone makes me worry whenever I think about this unpredictable epileptic attacks.
Interviewer	How do you go about stigma while caring for your person?
Respondent	*I seek advice and treatment from the health workers.*
Interviewer	*What do you think you can do as a result of the challenges you face while caring for a person with epilepsy?*
Respondent	*I talk to trusted people to give me an idea of what to do.*
Interviewer	*What can you do to a situation that calls for your absence from a person with epilepsy?*
Respondent	*Well…….. as I have told you I do not stay with her we only talk on phone and she tells me that if she is not feeling well her best friend helps her.*
Interviewer	*Families get affected while caring for a person with epilepsy, How is your family affected?*
Respondent	*We feel bad because we have tried all modes of treatment in vain now we do not know what to do. Now that she is old and staying away from home she goes for treatment alone I only pray for her because I have nothing to do.*

Case 19

SOCIAL BURDEN

Interviewer	Persons with epilepsy can get attacks (epileptic) at any time in any place, how do these attacks influence your public relationship?
Respondent	*[child crying] sometimes the attack comes when she has paid her friends a visit they usually help her to bring her back home, other people feel pity of me they say eeh!...you have a problem but they advise me to try treatment.*
Interviewer	At times these epileptic attacks come abruptly. How do you feel about the unpredictable epileptic attacks?
Respondent	It makes me worry and mere thought about it treats me bad, many times I think if she gets an attack near a water body or fire she may drown and die or get burnt by fire.
Interviewer	How do you go about stigma while caring for your person?
Respondent	*The truth is people say that this disease is demonic but they don`t care about their talks me I go ahead and take care of my person, I get for her drugs like I have come and we pray for her.*
Interviewer	*What do you think you can do as a result of the challenges you face while caring for a person with epilepsy?*
Respondent	*I take her for treatments as well as I pray for her.*
Interviewer	*What can you do to a situation that calls for your absence from a person with epilepsy?*
Respondent	*I leave to someone who can help her, I give her directions about the patients treatment then I go away if I have not traveled with my mother she keeps around with the patient and my sisters can also help.*
Interviewer	*Families get affected while caring for a person with epilepsy, How is your family affected?*
Respondent	*My wife and her siblings don`t feel bad ,they play with her and stay happily except when she gets an attack that is when they feel bad, but they take good care of her until she stabilizes.*

Case 20

SOCIAL BURDEN

Interviewer	Persons with epilepsy can get attacks (epileptic) at any time in any place, how do these attacks influence your public relationship?
Respondent	*[people talking from outside] it usually happens at school and people there tend to isolate him there was one particular teacher who could talk bad about him that would make my brother get hurt he would report to me and I just console him.*
Interviewer	At times these epileptic attacks come abruptly. How do you feel about the unpredictable epileptic attacks?
Respondent	For my case generally I worry because it`s unpredictable so you can`t know the consequences.
Interviewer	How do you go about stigma while caring for your person?
Respondent	*Me I don`t mind about peoples words, I just help him as a brother, some people tend to isolate him but me I can`t run away from him because the say blood is thinker than water since I know the truth about the disease and I also believe in God, one day my brother will be healed.*
Interviewer	*What do you think you can do as a result of the challenges you face while caring for a person with epilepsy?*
Respondent	*I pray and seek medical advice.*
Interviewer	*What can you do to a situation that calls for your absence from a person with epilepsy?*
Respondent	*When I am not at home, at least there has to be somebody who is close to him, I make sure that my phone is on so that I am notified if his condition changes there I call people who can help me to take him in the hospital.*
Interviewer	*Families get affected while caring for a person with epilepsy, How is your family affected?*
Respondent	*We feel desperate, for sure we have tried our level best but things are not working out so sometimes we feel worried.*

Case 21

SOCIAL BURDEN

Interviewer	Persons with epilepsy can get attacks (epileptic) at any time in any place, how do these attacks influence your public relationship?
Respondent	*Some help me and hold her but other people don't hold her.*
Interviewer	At times these epileptic attacks come abruptly. How do you feel about the unpredictable epileptic attacks?
Respondent	I feel worried that the attack may come and I fear that she may die like that.
Interviewer	How do you go about stigma while caring for your person?
Respondent	*Me I don't care in fact I continue getting for her medicine and take good care of her.*
Interviewer	*What do you think you can do as a result of the challenges you face while caring for a person with epilepsy?*
Respondent	*I seek medicine advise and treatment*
Interviewer	*What can you do to a situation that calls for your absence from a person with epilepsy?*
Respondent	*There sibling who can help her I leave her with them her followers, but they have grown up they cannot take care of her*
Interviewer	*Families get affected while caring for a person with epilepsy, How is your family affected?*
Respondent	*They don't feel fine, they feel bad because of their sister's illness they don't have peace.*

Case 22

SOCIAL BURDEN

Interviewer	Persons with epilepsy can get attacks (epileptic) at any time in any place, how do these attacks influence your public relationship?
Respondent	*It has happened like three times when she was at the trading centre when it came they don`t know that she has a problem, my mum knew what to do, they take her to the room and asked her what had happened she said I don`t know? They don`t know her condition and they saw begin like scratching.*
Interviewer	At times these epileptic attacks come abruptly. How do you feel about the unpredictable epileptic attacks?
Respondent	Really if it happens when she is preparing a meal I feel bad because you know may be she is boiling water then it comes that's why sometimes I tell my mum never to leave her alone.
Interviewer	How do you go about stigma while caring for your person?
Respondent	*I tried to inform my mum and Dady that this is not a clan thing and what people believe in is not true until now they have understood about this disease.*
Interviewer	*What do you think you can do as a result of the challenges you face while caring for a person with epilepsy?*
Respondent	*I talk to her about her condition, I health educate the rest, I ensure she gets drugs.*
Interviewer	*What can you do to a situation that calls for your absence from a person with epilepsy?*
Respondent	*When I am at school most of my time I leave her under the care of my Dady and mum but I help by picking drugs for her.*
Interviewer	*Families get affected while caring for a person with epilepsy, How is your family affected?*
Respondent	*May be my mum because she is the one taking care of her, there is no problem all of them are educated they know the problem they are able to support her.*

case 23

SOCIAL BURDEN

Interviewer	Persons with epilepsy can get attacks (epileptic) at any time in any place, how do these attacks influence your public relationship?
Respondent	*There are some people who rejoice others laugh at him and pass the other side when he has got an attack and then there are those people who feel pity, they help him and bring him home. many people think about this disease differently.*
Interviewer	At times these epileptic attacks come abruptly. How do you feel about the unpredictable epileptic attacks?
Respondent	I feel so bad because if it comes when he is in the kitchen or when he has gone to fetch water in fact I pray that attack may never come when he is in such places.
Interviewer	How do you go about stigma while caring for your person?
Respondent	*What keeps me strong is that I know this disease can get any other person, secondly other parents can produce three or more children suffering from this disease, but me I have only one child who is sick, I have accepted this situation and I know that it`s normal because other people have patients with Sickle cells, Cancer that make them get hospitalized from time to time but my boy is treated as an outpatient.*
Interviewer	*What do you think you can do as a result of the challenges you face while caring for a person with epilepsy?*
Respondent	*There is nothing that I can do except I maintain him on drugs, the only problem I know is that he has to shallow them until he dies.*
Interviewer	*What can you do to a situation that calls for your absence from a person with epilepsy?*
Respondent	*I leave him under Gods care because most of the time he disappears from home, however I also tell those people who remain at home to take care of him because it`s hard to keep him in one place unless you put a chain on him.*
Interviewer	*Families get affected while caring for a person with epilepsy, How is your family affected?*
Respondent	*They have pain because they had many expectations from him but his sickness has made him to stop studying.*

Case 2 [illegible]

SOCIAL BURDEN

Interviewer	Persons with epilepsy can get attacks (epileptic) at any time in any place, how do these attacks influence your public relationship?
Respondent	*I remember there is one time she fell at a funeral place most people feared and they did not get close to her but one person come and helped me to hold her then me made her to lie down in a safer place.*
Interviewer	At times these epileptic attacks come abruptly. How do you feel about the unpredictable epileptic attacks?
Respondent	I normally worry that this attack can throw her in a dangerous place like water or fire one day since he is a lady and she has to fetch water and cannot avoid cooking food especially when she gets married.
Interviewer	How do you go about stigma while caring for your person?
Respondent	*[mother laughs.......hahaha] I normally don`t mind about peoples ill talks, because I don`t know how this disease come about all I do is to pray for my daughter.*
Interviewer	*What do you think you can do as a result of the challenges you face while caring for a person with epilepsy?*
Respondent	*I pray to God.*
Interviewer	*What can you do to a situation that calls for your absence from a person with epilepsy?*
Respondent	*When I go away or some where she stays with her aunt.*
Interviewer	*Families get affected while caring for a person with epilepsy, How is your family affected?*
Respondent	*My family members don`t feel bad about her sickness, she has lived with this condition for 20 years we treat her well and give her the necessary support that she needs.*

Case ~~[illegible]~~ 25

SOCIAL BURDEN

Interviewer	Persons with epilepsy can get attacks (epileptic) at any time in any place, how do these attacks influence your public relationship?
Respondent	*The public relationship gets affected because I lack time to do any thing in public since most of the time I attend to him when he collapses in a public place I feel small in my relationship with people is affected some of them run away and leave me with the problem.*
Interviewer	At times these epileptic attacks come abruptly. How do you feel about the unpredictable epileptic attacks?
Respondent	I feel bad because it comes at any time when am not ready to do anything and this may cause more injury to the patient's body I feel bad and stressed at the same time.
Interviewer	How do you go about stigma while caring for your person?
Respondent	*Me as a person I keep on listening to gospel music and reading the bible this makes me to forget some things ,it reduces on my thoughts, I feel encouraged and I manage to cope with the situation.*
Interviewer	*What do you think you can do as a result of the challenges you face while caring for a person with epilepsy?*
Respondent	*When we are at the hospital I just have that hope that one day he will be okay.*
Interviewer	*What can you do to a situation that calls for your absence from a person with epilepsy?*
Respondent	*I make sure I don't take long to do something else other than caring for him unless there is another relative to back stop me.*
Interviewer	*Families get affected while caring for a person with epilepsy, How is your family affected?*
Respondent	*They get stigmatized within the community, people laugh at us and talk ill things about us,my brother has failed to marry. He even failed to complete his studies*

case 26

SOCIAL BURDEN

Interviewer	Persons with epilepsy can get attacks (epileptic) at any time in any place, how do these attacks influence your public relationship?
Respondent	*The time she fell in a public place, people ran away and went saying that disease is bad if you near the patient wind may blow the patient's saliva on you and you get the infection.*
Interviewer	At times these epileptic attacks come abruptly. How do you feel about the unpredictable epileptic attacks?
Respondent	I feel so bad when my daughter gets an attack because she can get injured or even die.
Interviewer	How do you go about stigma while caring for your person?
Respondent	*I become strong in my heart I don't answer people who talk ill, I talk to them very well and tell them I also don't know where this disease came from. I also encouraged the mother to be strong and don't mind about them because the patient is ours.*
Interviewer	*What do you think you can do as a result of the challenges you face while caring for a person with epilepsy?*
Respondent	*I take her to the hospital for treatment.*
Interviewer	*What can you do to a situation that calls for your absence from a person with epilepsy?*
Respondent	*I leave her with the mother and siblings when they come back from school they be with her*
Interviewer	*Families get affected while caring for a person with epilepsy, How is your family affected?*
Respondent	*As the family, we are not affected in any way it is the child her self after getting an attack she begins crying and tells the mother what can I do so that this sickness can leave me, she wishes that she could die .because this attacks come in a strong manner even after she has just swallowed the tablets.*

case 27

SOCIAL BURDEN

Interviewer	Persons with epilepsy can get attacks (epileptic) at any time in any place, how do these attacks influence your public relationship?
Respondent	*There are people who understand as parents they share the pain with me and they help when the boy is attacked but there are those people who say why he moves with a sick child and they just look on when he is attacked.*
Interviewer	At times these epileptic attacks come abruptly. How do you feel about the unpredictable epileptic attacks?
Respondent	It is not really good by the time the attack gets him he can fall anywhere even in dangerous places like in fire.
Interviewer	How do you go about stigma while caring for your person?
Respondent	*As for me I ignore them, I just look at them as they talk ill about my child and family.*
Interviewer	*What do you think you can do as a result of the challenges you face while caring for a person with epilepsy?*
Respondent	*I always make sure that I have money so that the patient is given whatever he needs at a specific time.*
Interviewer	*What can you do to a situation that calls for your absence from a person with epilepsy?*
Respondent	*I have to make sure that i inform somebody to help me in my absence.*
Interviewer	*Families get affected while caring for a person with epilepsy, How is your family affected?*
Respondent	*My family has no problem ,they love the boy.*

Case 28

SOCIAL BURDEN

Interviewer	Persons with epilepsy can get attacks (epileptic) at any time in any place, how do these attacks influence your public relationship?
Respondent	*At school they have refused her to study because they fear if she gets an attack nobody can help her so they decided to tell me let her remain at home. Sometimes there are people who can help sometimes they leave her there and I struggle alone.*
Interviewer	How do you feel about the unpredictable epileptic attacks?
Respondent	I have just left it to God he is the one to know because it just comes abruptly you cannot know so it is God to know.
Interviewer	How do you go about stigma while caring for your Sister?
Respondent	*Since I told you that I am the only person who is managing her, if people talk I don't mind because I don't have anybody to leave her with. I cannot throw her away, and I cannot chase her away from the family.*
Interviewer	*What do you think you can do as a result of the challenges you face while caring for a person with epilepsy?*
Respondent	*We go with her to church, at first people were thinking that it's the demons that attack her, they used to advise us to go to witch doctors we tried but all in vain it failed so from that time we decided to remain in God and we have an uncle who advised us to start using medication since then at least there is some change.*
Interviewer	*What can you do to a situation that calls for your absence from a person with epilepsy?*
Respondent	*When I am going far, I have a neighbor who helps me to look after her, only that because of the nature of this condition I always ensure that I travel back home I cant spent a night away from my home.*
Interviewer	*Families get affected while caring for a person with epilepsy, How is your family affected?*
Respondent	*My family has not been affected in any way, we cannot ignore her, we have nowhere to throw her away, and we work hand to hand to help her as a family.*

cax 29

SOCIAL BURDEN

Interviewer	Persons with epilepsy can get attacks (epileptic) at any time in any place, how do these attacks influence your public relationship?
Respondent	*Sometimes there are people who can help sometimes they leave her there and I struggle alone.*
Interviewer	How do you feel about the unpredictable epileptic attacks?
Respondent	I feel so bad because the attacks just come abruptly I wish I knew when it will occur I would take precautions.
Interviewer	How do you go about stigma while caring for your Sister?
Respondent	*I have managed to cope with stigma because I don't mind what people say about me and I have continued to take care of my child the way doctors advise me from the hospital.*
Interviewer	*What do you think you can do as a result of the challenges you face while caring for a person with epilepsy?*
Respondent	*The way I manage is by going with her to the church, at first people used to advise us to go to witch doctors we even tried them but all in vain my daughter has not cured.*
Interviewer	*What can you do to a situation that calls for your absence from a person with epilepsy?*
Respondent	*When I am going away I leave her under the care of her siblings, they have all learnt how to manage her when she gets an attack.*
Interviewer	*Families get affected while caring for a person with epilepsy, How is your family affected?*
Respondent	*My family has not been affected in any way we support her as a family.*

Case 30

SOCIAL BURDEN

Interviewer	Persons with epilepsy can get attacks (epileptic) at any time in any place, how do these attacks influence your public relationship?
Respondent	*[People talking from outside] usually when it happens people tend to isolate themselves from us. Some people talk bad words about us.*
Interviewer	At times these epileptic attacks come abruptly. How do you feel about the unpredictable epileptic attacks?
Respondent	I worry because it`s unpredictable so you can`t know the consequences the child may get injured or even die.
Interviewer	How do you go about stigma while caring for your person?
Respondent	*Me I don`t mind about peoples words, I just help him as a parent.*
Interviewer	*What do you think you can do as a result of the challenges you face while caring for a person with epilepsy?*
Respondent	*I pray and seek medical advice.*
Interviewer	*What can you do to a situation that calls for your absence from a person with epilepsy?*
Respondent	*When I am not at home, I make sure that my phone is on so that I am notified if the condition changes, I leave for them money for emergency and make sure that my wife stays at home.*
Interviewer	*Families get affected while caring for a person with epilepsy, How is your family affected?*
Respondent	*We feel sad, we have tried our level best but things are not working out we feel worried.*
