# Supplementary material for: Lived experiences of caregivers of persons with epilepsy attending an epilepsy clinic at a tertiary hospital, eastern Uganda: A phenomenological approach
Source: PLoS One. 2023 Jul 18;18(7):e0274373. doi: 10.1371/journal.pone.0274373 (PMC10353802; doi:10.1371/journal.pone.0274373)
Supplement: S1 Data — (ZIP) [file pone.0274373.s001.zip › Physical burden.pdf]

Case 1.

Physical burden

Interviewer	As a care giver you really need to attend to these patients and give in more of your time. How do you find the care giving responsibility?
Respondent	It's not an easy job, my child doesn't like eating I feed her by force, and I also lack money to buy food at times.
Interviewer	I know as you have said care giving is not an easy job, what physical challenges do you face that may affect your health?
Respondent	I have to carry the baby most of the time I get fatigued sometimes when I think a lot about my daughter illness I develop headache.
Interviewer	In deed caring for a person with epilepsy is tiresome now I would like to know how about at night when you also need to have rest what time do you always get your sleep?
Respondent	I sleep well without any disturbance I only wake up in the night to feed her.
Interviewer	Ok.... I would like to know how you feed yourself while you have this burden of caring for your daughter.
Respondent	When the attack comes I don't feed at all but when the baby is fine, I eat 3 meals in a day though sometimes I lack appetite for food.

Case 2

Physical burden

Interviewer	As a care giver you really need to attend to your sister and give in more of your time. How do you find the care giving responsibility?
Respondent	I have no problem with it because I want her to get fine.
Interviewer	What physical challenges do you face while caring for your sister that may affect your health?
Respondent	[Interruption phone ringing] I get fatigued sometimes my head aches.
Interviewer	I know caring for a person with epilepsy is tiresome now I would like to know how about at night when you also need to have rest what time do you always get your sleep?
Respondent	When she has had an attack during day time I don't sleep well because I fear that the attack may come again.
Interviewer	I would like to know how you feed yourself while you have this hard task of caring for your sister.
Respondent	At times I get so worried and fail to eat.

Case 3

Physical burden

Interviewer	As a care giver, How do you find the care giving responsibility?
Respondent	Sometimes I say it's God who planned it that way even if I talk, even if I kick her or do what, it doesn't help I just have to take care of her.
Interviewer	What physical challenges do you face while caring for your sister that may affect your health?
Respondent	I experience chest pain sometimes I also get headache
Interviewer	I would like to know how about at night when you need to rest what time do you always get sleep.
Respondent	I sleep well I am not disturbed at night, those attacks only come during daytime
Interviewer	I would like to know how you feed yourself while you have this hard task of caring for your daughter.
Respondent	Sometimes she can disturb me and I end up losing appetite or missing meals all my minds be on her when she fits.

Case 4

Physical burden

Interviewer	As a care giver, How do you find the care giving responsibility?
Respondent	He disturbs but I have nothing to do because I am his mother if I leave him there who will look after him, he is my fifth born but I take care of him more than the rest I tell them this one is sick they also understand.
Interviewer	What physical challenges do you face while caring for your son that may affect your health?
Respondent	Those days he used to disturb me i would bring him to the hospital while beating him, we would fight on the way and I could go back home when I am tired, feeling chest pain and backache.
Interviewer	I would like to know how about at night when you need to rest what time do you always get sleep.
Respondent	We sleep well at night; he normally fits in the morning and during daytime.
Interviewer	I would like to know how you feed yourself while you have this hard task of caring for your son.
Respondent	When my son is okay I eat 3 meals in a day because I have nothing to worry about but if I was going to eat and I notice that he is about to throw fit I can't eat because he can stay the whole day in that state without eating food or taking any sdrink.

Case 5

Physical burden

Interviewer	As a care giver, How do you find the care giving responsibility?
Respondent	It's difficult sometimes you can think that he is playing with the neighbor's children but when you go to look for him they tell you that he has gone somewhere else, he doesn't like bathing so you have to force him to bathe he keeps on dodging and when he is tired he just pours water on his body.
Interviewer	What physical challenges do you face while caring for your grandson that may affect your health?
Respondent	I don't have any.
Interviewer	I would like to know how about at night when you need to rest what time do you always get sleep.
Respondent	I sleep well except when he gets an attack at night there you can't sleep completely you have to keep monitoring him.
Interviewer	I would like to know how you feed yourself while you have this hard task of caring for your son.
Respondent	When he is okay we eat, laugh and enjoy food but when he is attacked Aaah……….. you can't eat and enjoy.

Case 6

Economic burden

Interviewer	How does caring for your husband interfere with your source of income?
Respondent	Being a farmer when I lack transport to take him to hospital I sell the little maize met for food and get money for our use but we end up lacking food to eat.
Interviewer	As a caregiver you need money while caring for your husband do you have problems with money?
Respondent	Like I said earlier on that I don't work, I have to feed my family, I have to pay school fees for my children and cater for my husband's treatment single handedly. I end up using all the money and I lack!
Interviewer	How does caring for your husband affect your productivity at work?
Respondent	Whenever I start new business, my husband gets serious attacks and it becomes difficult for me to take care of him and manage my business at the same time. I end up losing customers and using all the money meant for sustaining my business.
Interviewer	I know that you spend much of your time caring for the patient and most of the time we struggle to make our finances stable what do you think can happen to your financial status?
Respondent	Money can get over and you become poor.

Case 7

Economic burden

Interviewer	How does caring for your daughter interfere with your source of income?
Respondent	I have to get up very early and go to the market for shopping but I have failed to manage because my daughter and hospitalized from time to time.
Interviewer	As a caregiver you need money while caring for your daughter do you have problems with money?
Respondent	I don't have money right now I used to work those days I used to have money but it got done
Interviewer	How does caring for your daughter affect your productivity at work?
Respondent	When she is fine you can go with her and she sits at your work place while you work. but now that she is sick you can't leave her alone
Interviewer	I know that you spend much of your time caring for the patient and most of the time we struggle to make our finances stable what do you think can happen to your financial status?
Respondent	You worry because you don't have what to eat you have to treat up the patient you need to dress well and look smart but all these are not there I feel so bad because I know how to do business but I can not leave my daughter alone.0101

Case 8

Economic burden

Interviewer	How does caring for your daughter interfere with your source of income?
Respondent	For us here in the village we don`t have money all the time sometimes we can work and get money which helps the family, if you have not got money your family can not have what to eat and even the patient does not receive treatment.
Interviewer	As a caregiver you need money while caring for your daughter do you have problems with money?
Respondent	We earn through digging sometimes we hire land for tilling but this dry season we remained without money, we have sold goats, fire wood, chicken but all the money we receive goes for treatment of our daughter, you cannot keep money when your child is sick, a child is the heart of every parent.
Interviewer	How does caring for your daughter affect your productivity at work?
Respondent	I cannot work well I worry about the patient's condition when I leave her behind because she is sick you cannot be energetic enough to work.
Interviewer	I know that you spend much of your time caring for the patient and most of the time we struggle to make our finances stable what do you think can happen to your financial status?
Respondent	You can develop diseases like pressure, ulcers because all the time you are worried and thinking in your heart you don`t think of anything good you only think about sickness when you look at your situation and you look at the patient's condition in your heart you say what should I do for my daughter so that she can get well.

Case 9

Physical burden

Interviewer	As a care giver, How do you find the care giving responsibility?
Respondent	It is hard, you worry a lot your heart all the time is worried and fear that she might fall and injure herself.
Interviewer	What physical challenges do you face while caring for your daughter that may affect your health?
Respondent	The back aches, I get fatigued and I have many thoughts.
Interviewer	I would like to know how about at night when you need to rest what time do you always get sleep.
Respondent	As you go to sleep the first sleep catches me but the second one I sleep less because of worrying.
Interviewer	I would like to know how you feed yourself while you have this hard task of caring for your daughter.
Respondent	We eat but when she gets an attack we don`t eat I keep on worrying that this sickness may kill her at any time.

Case 10

Physical burden

Interviewer	As a care giver, How do you find the care giving responsibility?
Respondent	It is heavy because when I am with the patient I have no time to do what can sustain me or get money to get what I want.
Interviewer	What physical challenges do you face while caring for your son that may affect your health?
Respondent	I feel my brain is not so good I lack energy the situation is not good.
Interviewer	I would like to know how ~~about~~ at night when you need to rest what time do you always get sleep.
Respondent	I sleep little but I am not all that ok to sleep, I think how will I be tomorrow, how will I take care of my patient and others as you have seen.
Interviewer	I would like to know how you feed yourself while you have this hard task of caring for your daughter.
Respondent	I have less appetite I have thoughts of sickness sometimes I try to eat sometimes appetite goes.

Case 1

Physical burden

Interviewer	As a care giver, How do you find the care giving responsibility?
Respondent	It is hard because like me who is employed you are working but they are giving you other responsibilities but when it becomes to this one you are called officially, you man you take your person we cannot manage so that means you have to leave the responsibility the other side, finish that thing but in side you, you are saying you mean you cannot even help me but they don`t listen.
Interviewer	What physical challenges do you face while caring for your son that may affect your health?
Respondent	Because we over talk and the person is not understanding you, you end up being stressed and develop headache.
Interviewer	I would like to know how about at night when you need to rest what time do you always get sleep.
Respondent	At night you may not sleep when somebody is disturbing you by being attacked so you also have to be there, you cannot leave her alone because she can get injured you have to stay away and you watch over her.
Interviewer	I would like to know how you feed yourself while you have this hard task of caring for your daughter.
Respondent	I don`t eat most of the times because I may not have prepared food at home especially when I am alone or I may be taken up caring for her and end up missing meals.

Case 12

Physical burden

Interviewer	As a care giver, How do you find the care giving responsibility?
Respondent	It`s heavy, you have to feed him, you can`t go anywhere, and you can`t do other personal things.
Interviewer	What physical challenges do you face while caring for your son that may affect your health?
Respondent	I get headache after thinking so much.
Interviewer	I would like to know how about at night when you need to rest what time do you always get sleep.
Respondent	If he gets attacked I can`t sleep, he bits his tongue sometimes he can fall three to four times and I cannot sleep during day time and I keep awake at night to monitor him.
Interviewer	I would like to know how you feed yourself while you have this hard task of caring for your daughter.
Respondent	When he gets an attacked, there after he settles down then I feed him but myself I can`t get the energy to eat but if he is fine I feel settled and I can eat well.

Case 13

Physical burden

Interviewer	As a care giver, How do you find the care giving responsibility?
Respondent	It is not easy I reached somewhere and I got tired and asked God to strengthen me and I regained strengths.
Interviewer	What physical challenges do you face while caring for your son that may affect your health?
Respondent	In most cases I experience headache, I can spend the whole week feeling headache.
Interviewer	I would like to know how about at night when you need to rest what time do you always get sleep.
Respondent	When he is sick he disturbs a lot I don't sleep at night.
Interviewer	I would like to know how you feed yourself while you have this hard task of caring for your daughter.
Respondent	I don't eat, I be there seated without energy I even don't work and I tell God to help me.

Case 14

Physical burden

Interviewer	As a care giver, How do you find the care giving responsibility?
Respondent	It is not all that burdening me because as per now I don't have a child and it's not like I am having a family if I was married I would have many things to think about and many thing to do but now my concern is on her.
Interviewer	What physical challenges do you face while caring for your son that may affect your health?
Respondent	No everything is okay with me.
Interviewer	I would like to know how about at night when you need to rest what time do you always get sleep.
Respondent	I sleep well at nigh but if she is attacked during day time, I sleep but I don't give myself to that much sleep.
Interviewer	I would like to know how you feed yourself while you have this hard task of caring for your person.
Respondent	My eating habit is normal and okay.s

Case 15

Physical burden

Interviewer	As a care giver, How do you find the care giving responsibility?
Respondent	At times you feel like getting exhausted he gives a negative response he can refuse to eat on time before getting medication, at times when he gets an attack you think may be because he didn`t take his drugs.
Interviewer	What physical challenges do you face while caring for your son that may affect your health?
Respondent	No except for my own health because he helps himself most of the time a part from washing his clothes recently I just asked for water he looked for all his dirty clothes and started cleaning them.
Interviewer	I would like to know how about at night when you need to rest what time do you always get sleep.
Respondent	That's also the worry or challenge we have because the way our home is, we are not able to be in the same house and it has ever happened twice when he is there and he had closed the door but I tell him never lock the door but he had locked we failed to open and stood outside with my husband until morning. I sleep most of the times and I am at least in case that attacks him at night I can know but the thing of closing the door I have tired it.
Interviewer	I would like to know how you feed yourself while you have this hard task of caring for your person.
Respondent	Ahaa……. laughing this is my normal weight but it`s not long I have told you I just started since last year.

Case 16

Physical burden

Interviewer	As a care giver, How do you find the care giving responsibility?
Respondent	It`s not simple, it's very hectic I am somebody who has not been used to staying in one place but now my life style has changed, sitting in one place makes me more tired.
Interviewer	What physical challenges do you face while caring for your Dady that may affect your health?
Respondent	Yaah!... the headache is there all the time am thinking sometimes I feel as if I am getting ulcers.
Interviewer	I would like to know how about at night when you need to rest what time do you always get sleep.
Respondent	I am pre-occupied with thoughts I hardly get sleep sometimes especially when dates for reviews come and you don`t have money. Since they also say that the disease is genetic I sometimes think that it may show up in other family members and I imagine how I will handle the situation.
Interviewer	I would like to know how you feed yourself while you have this hard task of caring for your person.
Respondent	Normally when I think a lot all the time I am worried I don`t eat.

Case 17

Physical burden

Interviewer	As a care giver, How do you find the care giving responsibility?
Respondent	It is difficult, because your tied-up in one place you can't move anywhere all you have to do to take care of the patient
Interviewer	What physical challenges do you face while caring for your son that may affect your health?
Respondent	Me myself, I have hernia, but I have failed to undergo an operation due to lack of money when I think a lot about my illness and my sons condition I develop headache.
Interviewer	I would like to know how about at night when you need to rest what time do you always get sleep.
Respondent	I don`t sleep at all because of thoughts, when I try to catch some sleep the attack usually comes and I end up keeping awake the whole night.
Interviewer	I would like to know how you feed yourself while you have this hard task of caring for your person.
Respondent	My appetite goes because of thoughts whenever I get thoughts I don`t eat.

Case 18

Physical burden

Interviewer	As a care giver, How do you find the care giving responsibility?
Respondent	It is difficult, because you spend a lot and you have many thoughts all the time you keep asking yourself what can I do.
Interviewer	What physical challenges do you face while caring for your son that may affect your health?
Respondent	I am stressed most of the time because of thinking so much.
Interviewer	I would like to know how about at night when you need to rest what time do you always get sleep.
Respondent	I normally sleep but when thoughts about this disease come I lose sleep.
Interviewer	I would like to know how you feed yourself while you have this hard task of caring for your person.
Respondent	My appetite is okay but when my daughter calls and tells me that she got an attack I lose appetite for food.

Case 19

Physical burden

Interviewer	As a care giver, How do you find the care giving responsibility?
Respondent	Ahaa!....it is heavy, what brings that is you have to be near the patient not allowing her to go far and you can`t also go far, that makes it difficult task.
Interviewer	What physical challenges do you face while caring for your daughter that may affect your health?
Respondent	I get so tired because I have to hold her with the lot of energy and even those attacks can come three times a day and she becomes violent.
Interviewer	I would like to know how about at night when you need to rest what time do you always get sleep.
Respondent	I don`t sleep well, I worry about those attacks that come at night.
Interviewer	I would like to know how you feed yourself while you have this hard task of caring for your person.
Respondent	I don`t eat well in fact I eat but I don`t get satisfied because of lots of worries.

Case 20

Physical burden

Interviewer	As a care giver, How do you find the care giving responsibility?
Respondent	It is a little bit hard because epileptic patient is not like any other patient because when he develops an attack you have to make sure that you support him so that he doesn't get an injury of which sometimes he can prove to be much stronger than you it is a little hard but you just have to endure and move on.
Interviewer	What physical challenges do you face while caring for your daughter that may affect your health?
Respondent	No, usually not because when he develops those attacks it is difficult to manage him alone by myself.
Interviewer	I would like to know how about at night when you need to rest what time do you always get sleep.
Respondent	Initially the attacks would come mostly at night I hard to wake up in the night at around 2:00am you have to struggle to rush him to hospital to get treatment it can be very frustrating especially at night in the midst of the night you feel you are trapped as if you are helpless.
Interviewer	I would like to know how you feed yourself while you have this hard task of caring for your person.
Respondent	When he gets the attack I usually for get about food and concentrate on him when giving the care.

Case 21

Physical burden

Interviewer	As a care giver, How do you find the care giving responsibility?
Respondent	Its heavy because all the time you have to be where she is.
Interviewer	What physical challenges do you face while caring for your daughter that may affect your health?
Respondent	I feel general body pains and fatigue.
Interviewer	I would like to know how about at night when you need to rest what time do you always get sleep.
Respondent	I don't get sleep very well because I know the attack can come at night any time
Interviewer	I would like to know how you feed yourself while you have this hard task of caring for your person.
Respondent	Ahaa... I don't eat well, my heart vouches somewhere and I lack appetite because I have many thoughts.

case 22

Physical burden

Interviewer	As a care giver, How do you find the care giving responsibility?
Respondent	It`s bad, it is difficult because it`s not a short time duration, I think it will take the rest of someone's life and also the care has to be there ,it`s a chronic illness that will take some time so someone has to have enough time.
Interviewer	What physical challenges do you face while caring for your daughter that may affect your health?
Respondent	I get fatigue but just for a short time so for it has happened 3-4 times when it happens fatigue must come to because she is heavy.
Interviewer	I would like to know how about at night when you need to rest what time do you always get sleep.
Respondent	She sleeps with my mum but she has to fear
Interviewer	I would like to know how you feed yourself while you have this hard task of caring for your person.
Respondent	The way someone behaves makes you lose appetite they can`t bring for you food when someone is tackling herself everywhere you lose appetite.

Case 23

Physical burden

Interviewer	As a care giver, How do you find the care giving responsibility?
Respondent	You may cry that you have an epileptic patient but you find someone with patient suffering from sickle cells who is with hospitalized in a weekly basis, as you are still there, another one runs mad, and the mad one gets worse you will even say at least this is epileptic patient, he may fall for two minutes and gets well but a mad one gets rubbish and eat, when you see this happening you become strong and be there.
Interviewer	What physical challenges do you face while caring for your daughter that may affect your health?
Respondent	No I don`t experience any physical complains or challenges.
Interviewer	I would like to know how about at night when you need to rest what time do you always get sleep.
Respondent	You can`t sleep because every ones life needs peace and joy if you have issues that disturb you in life you can`t sleep, it's not good for someone to fall sick though it`s a natural happening and we have no control over it.
Interviewer	I would like to know how you feed yourself while you have this hard task of caring for your person.
Respondent	You can`t get appetite if your to eat you may eat very little food sometimes you just go and sleep hungry.

Case 24

Physical burden

Interviewer	As a care giver, How do you find the care giving responsibility?
Respondent	Well, when you have money to buy drugs the responsibility is lighter, but if you lack money it`s hard.
Interviewer	What physical challenges do you face while caring for your daughter that may affect your health?
Respondent	I do not experience any physical illness as a result of my care giving responsibility.
Interviewer	I would like to know how about at night when you need to rest what time do you always get sleep.
Respondent	Me I sleep well except when she gets an attack there I can`t sleep, I keep monitoring her the whole night to see because another attack may come again.
Interviewer	I would like to know how you feed yourself while you have this hard task of caring for your person.
Respondent	When she is attacked, I first wait for her to stabilize that is when I eat food if she does not stabilize my interest of food goes away.

Case 25

Physical burden

Interviewer	As a care giver, How do you find the care giving responsibility?
Respondent	Really its not easy its a big challenge whereby I just have to sit when I am ready for anything that may happen to the patient.
Interviewer	What physical challenges do you face while caring for your daughter that may affect your health?
Respondent	Yes, at times I have to carry him and he is heavy in this process I end up feeling chest pain, backache, I really become tired especially when he collapses it's a big burden to me.
Interviewer	I would like to know how about at night when you need to rest what time do you always get sleep.
Respondent	Ahaa... I don't have a specific time of sleeping because any time the patient can get an attack. I just rest for some few minutes but I don't sleep deeply. My sleeping habit keeps on fluctuating, depending on the patient's condition if is fine I sleep well if he gets an attack I have to keep awake and monitor his condition.
Interviewer	I would like to know how you feed yourself while you have this hard task of caring for your person.
Respondent	Its hard sometimes when I am eating and he gets an attack I loose appetite for food.

Case 26

Physical burden

Interviewer	As a care giver, How do you find the care giving responsibility?
Respondent	It is hard, you have to have money because buying everything needs money, if you don't have money a child cannot be alive.
Interviewer	What physical challenges do you face while caring for your daughter that may affect your health?
Respondent	For my child's seek I cannot get tired there is no physical challenge that I get while caring for her.
Interviewer	I would like to know how about at night when you need to rest what time do you always get sleep.
Respondent	At night we sleep together on the same bed because she is still young we normally sleep well the attacks she gets only come during day time.
Interviewer	I would like to know how you feed yourself while you have this hard task of caring for your person.
Respondent	I feed well, but when she gets an attack, the food loses the taste I just cannot eat when my daughter is not well.

case 27

Physical burden

Interviewer	As a care giver, How do you find the care giving responsibility?
Respondent	I have no problem with taking care of my own son.
Interviewer	What physical challenges do you face while caring for your daughter that may affect your health?
Respondent	No, usually not because when he develops those attacks it is difficult to manage him alone by myself.
Interviewer	I would like to know how about at night when you need to rest what time do you always get sleep.
Respondent	I sleep well but when the attack comes I sleep next to the child i sleep but I don't sleep soundly so I have to be alert.
Interviewer	I would like to know how you feed yourself while you have this hard task of caring for your person.
Respondent	I eat well, my appetite for food is there but when the attack comes the appetite automatically goes away when he is sick I don't feel all that fine food doesn't enter.

case 28

Physical burden

Interviewer	As a care giver, How do you find the care giving responsibility?
Respondent	It is not easy because the time you are supposed to do other things you spend it attending to this patient because of the fear of leaving her alone wherever you go you must move with her because of her delicate life.
Interviewer	What physical challenges do you face while caring for your daughter that may affect your health?
Respondent	Sometimes I feel chest pain because she is heavy as you see her body.
Interviewer	I would like to know how about at night when you need to rest what time do you always get sleep.
Respondent	Sometimes at night I don`t because this thing is like a demon it can come and squeeze her in the middle of the night it becomes terrible you can`t sleep.
Interviewer	I would like to know how you feed yourself while you have this hard task of caring for your person.
Respondent	Yes I eat well, but if an attack comes I automatically leave food and attend to her.

case 29

Physical burden

Interviewer	As a care giver, How do you find the care giving responsibility?
Respondent	It is not easy because the time you are supposed to do other things you spend it attending to the patient .
Interviewer	What physical challenges do you face while caring for your daughter that may affect your health?
Respondent	Nurse mostly I develop headache because I have endless thoughts..
Interviewer	I would like to know how about at night when you need to rest what time do you always get sleep.
Respondent	Sometimes at night I don`t sleep because this thing can decide to come at any time if you choose to sleep your person can fall off from the bed, injure herself badly or even die whenever I think of such I don't sleep.
Interviewer	I would like to know how you feed yourself while you have this hard task of caring for your person.
Respondent	I eat all meals 3 times in a day when my daughter is fine, but if an attack comes I automatically leave food and attend to her.

Case. 30

Physical burden

Interviewer	As a care giver, How do you find the care giving responsibility?
Respondent	It is hard because on top of looking after her I also have other children to take care of.
Interviewer	What physical challenges do you face while caring for your daughter that may affect your health?
Respondent	I usually develop terrible headache and i don't sleep at night I keep thinking, honestly my daughter's sickness has disturbed me.
Interviewer	I would like to know how about at night when you need to rest what time do you always get sleep.
Respondent	I have become like a watchman me I don't sleep at night I don't want to lose my child when I am sleeping that attack comes even at night.
Interviewer	I would like to know how you feed yourself while you have this hard task of caring for your person.
Respondent	When she gets the attack I usually forget about food and concentrate on her but when she is fine I can eat something.
