# Supplementary material for: Lived experiences of caregivers of persons with epilepsy attending an epilepsy clinic at a tertiary hospital, eastern Uganda: A phenomenological approach
Source: PLoS One. 2023 Jul 18;18(7):e0274373. doi: 10.1371/journal.pone.0274373 (PMC10353802; doi:10.1371/journal.pone.0274373)
Supplement: S1 Data — (ZIP) [file pone.0274373.s001.zip › Economic burden 1.pdf]

Case 1

Economic burden

Interviewer	You know sometimes we have to look for money, you have to go to the garden, you have to work so that you earn a living how does caring for your person with epilepsy interfere with your source of income?
Respondent	I tried to work but I could not be settled at the work place and ended up leaving work because I could not leave her alone.
Interviewer	Ok.... Sorry about that now that you faced challenges at the work place and even ended up leaving work to care for her what other challenges do you face while caring for your daughter?
Respondent	Mmh..... Caring for her is expensive in terms of feeding sometimes I need to buy milk, sugar, millet flour mixed with soya beans yet I lack money.
Interviewer	To be productive at work one needs to have humble time and a settled mind, how does caring for a person with epilepsy affect your productivity at work?
Respondent	Well, I used to worry a lot and I would feel unsettled whenever I was away from my daughter this made me to stop working
Interviewer	Well I know that you spend much of your time caring for the patient and most of the time we struggle to make our finances stable what do you think can happen to your financial status?
Respondent	Ok....... Time will come and I lack money because of spending on feeding and medication.

Case 2

Economic burden

Interviewer	You know we have to look for money, you have to go to the garden, and you have to attend to your business so that you earn a living how does caring for your person with epilepsy interfere with your source of income?
Respondent	I have a small groccery shop that sells tomatoes, bananas and cabbages I also do farming that's how I get money but when she falls sick my business and garden work is affected because I have no one I can ask to take care of her.
Interviewer	As a caregiver you need money to buy food, buy drugs when the hospital cannot provide, and transport her to the hospital for review do you have problems with money?
Respondent	Yes sometimes i lack money .when I get fixed up I call my dad and ask him for money, when he has he gives us if he doesn't have we borrow and remain in debts.
Interviewer	To be productive at work one needs to have humble time and a settled mind, how does caring for a person with epilepsy affect your productivity at work?
Respondent	I have lost customers and used up all the money on caring for the patient.
Interviewer	I know that you spend much of your time caring for the patient and most of the time we struggle to make our finances stable what do you think can happen to your financial status?
Respondent	Ok……. This sickness has drained me financially, my business collapsed it's my dad who sends us help.

Case 3

Economic burden

Interviewer	You being a tailor I know you earn by making peoples clothes and satisfying your customers, how does caring for your daughter interfere with your source of income?
Respondent	It really affects me sometimes I don't work especially when the child is sick she needs much attention, when I haven't worked I don't get money also some customers quarrel at me and I lose them.
Interviewer	As a caregiver you need money to buy food, buy drugs when the hospital cannot provide, and transport her to the hospital for review do you have problems with money?
Respondent	Yes I have less time to work but I spend a lot of money on her treatment .I am forced to borrow capital to run my business from Sacco's and struggle to pay back.
Interviewer	To be productive at work one needs to have humble time and a settled mind, how does caring for a person with epilepsy affect your productivity at work?
Respondent	I have lost most of my customers and used up a lot of money because of my daughters illness now I am in debts.
Interviewer	I know that you spend much of your time caring for the patient and most of the time we struggle to make our finances stable what do you think can happen to your financial status?
Respondent	It can affect my payment of money back to the Sacco and I can't operate my business.

Case 4

Economic burden

Interviewer	How does caring for your son interfere with your source of income?
Respondent	I don't work but my husband is a business man he sells maize then we get money.
Interviewer	As a caregiver you need money while caring for your son do you have problems with money?
Respondent	Sometimes I lack money .If the appointment date reaches and I don't have money I make sure that I borrow and I bring him to the Hospital.
Interviewer	To get better yields from the garden you have to spent good time and invest money as well. How does caring for your son affect your productivity at work?
Respondent	It's from farming that I get food and money when I sell my harvests. Mmmh…… when you are taking care of a person like this you cannot leave him for long or even go far I dig a little and for a short while then I get back home that affects my productivity.
Interviewer	I know that you spend much of your time caring for the patient and most of the time we struggle to make our finances stable what do you think can happen to your financial status?
Respondent	You can become poor because the patient becomes your first priority you cannot keep money when you have a sick child.

Case 5

Economic burden

Interviewer	How does caring for your son interfere with your source of income?
Respondent	My source of income is the garden I have enough time if we go to the garden we walk with him it's just near home I don't get any inconvenience.
Interviewer	As a caregiver you need money while caring for your grandson do you have problems with money?
Respondent	I have problems with money because I don't work when drugs lack at the hospital and I am told to go and buy I just go back and sit because there is no money to buy
Interviewer	To get better yields from the garden you have to spent good time and invest money as well. How does caring for your grandson affect your productivity at work?
Respondent	Right now we are not digging or working it's a dry season our work is to fetch water, wash plates, cook food and sit at home my productivity is not affected.
Interviewer	I know that you spend much of your time caring for the patient and most of the time we struggle to make our finances stable what do you think can happen to your financial status?
Respondent	If I get that tension I will not get anything there will be no food, no digging, because now like a farmer when you are growing some little things when you harvest you get something from there you sell eggs ,beans and manage some responsibility.

Case 6

Physical burden

Interviewer	As a care giver, How do you find the care giving responsibility?
Respondent	Its not easy its difficult but I do it because there is nothing to do.
Interviewer	What physical challenges do you face while caring for your husband that may affect your health?
Respondent	I feel headache sometimes I also feel dizzy.
Interviewer	I would like to know how about at night when you need to rest what time do you always get sleep.
Respondent	I don't get enough sleep I always think about how I will feed the family, where I will get transport to go back to the hospital and how I will pay school fees for my children
Interviewer	I would like to know how you feed yourself while you have this hard task of caring for your husband.
Respondent	Appetite goes you cannot eat when he is attacked

Case 7

Physical burden

Interviewer	As a care giver, How do you find the care giving responsibility?
Respondent	It's difficult sometimes you can think that he is playing with the neighbor's children but when you go to look for him they tell you that he has gone somewhere else, he doesn't like bathing so you have to force him to bathe he keeps on dodging and when he is tired he just pours water on his body.
Interviewer	What physical challenges do you face while caring for your grandson that may affect your health?
Respondent	I don't have any.
Interviewer	I would like to know how about at night when you need to rest what time do you always get sleep.
Respondent	I sleep well except when he gets an attack at night there you can't sleep completely you have to keep monitoring him.
Interviewer	I would like to know how you feed yourself while you have this hard task of caring for your son.
Respondent	When he is okay we eat, laugh and enjoy food but when he is attacked Aaah………… you can't eat and enjoy.

Case 8

Physical burden

Interviewer	As a care giver, How do you find the care giving responsibility?
Respondent	It is a heavy job, you have to fetch for her water for bathing, prepare for her tea, wash all her clothe and ensure that she is smart all the time she has to be clean all the time.
Interviewer	What physical challenges do you face while caring for your grandson that may affect your health?
Respondent	You may not sleep during day time you sleep at night for few hours because you have to keep on checking on her so you end up getting headache and fatigue.
Interviewer	I would like to know how about at night when you need to rest what time do you always get sleep.
Respondent	You don`t sleep well, you keep waking up to check on the patient because all the time you are worried you may even fail to sleep.
Interviewer	I would like to know how you feed yourself while you have this hard task of caring for your daughter.
Respondent	You don`t eat well the stomach is in side all the time all the time you are worried even if you put your trust in God still you remain in thoughts.

Case 9

Economic burden

Interviewer	How does caring for your daughter interfere with your source of income?
Respondent	In the village things are like that we get little money and it helps us you can sell a goat and get money for use, we took her to the hospital and she got medicine so we are with her like that there is nothing to do.
Interviewer	As a caregiver you need money while caring for your daughter do you have problems with money?
Respondent	Yes we get some challenges some of our children are studying but there is no money when we go to the hospital and find that drugs are lacking we are forced to sell something at home and then buy medicine for her.
Interviewer	How does caring for your daughter affect your productivity at work?
Respondent	My daughter only sits there at home she does not interfere with our work we leave her and go the garden and when we have finished to dig we come back home and cook food we only worry when she gets an attack.
Interviewer	I know that you spend much of your time caring for the patient and most of the time we struggle to make our finances stable what do you think can happen to your financial status?
Respondent	[interruption child crying out side] Eeh!.........Eeh! it is a problem because when you do not have money then you can not have anything what do you.

Case 10

Economic burden

Interviewer	How does caring for your son interfere with your source of income?
Respondent	My earning is less because I don`t have time to do business all the time I have to keep my son.
Interviewer	As a caregiver you need money while caring for your son do you have problems with money?
Respondent	Yes because I don`t have enough time to work even what I do doesn`t progress well because I don`t have enough time to put in my best.
Interviewer	How does caring for your son affect your productivity at work?
Respondent	I can`t work well, I spent all the time caring for the patient and all my thought are always on him. I tried working but I could not settle at the work place.
Interviewer	I know that you spend much of your time caring for the patient and most of the time we struggle to make our finances stable what do you think can happen to your financial status?
Respondent	I will become poor because all I have goes to the patients feeding,transport and drugs.

Case 11

Economic burden

Interviewer	How does caring for your son interfere with your source of income?
Respondent	Because now all your mind is with this person you are not relaxed to think about digging,farming,opening other businesses you only focus on one thing that is getting salary yet there are many problems.
Interviewer	As a caregiver you need money while caring for your son do you have problems with money?
Respondent	Yaah.........you are helpless like now this one has come without any thing I am to buy food for her, buy drags for her even her family members have dis owned her now for me whatever happens financially is my problem I have owned her on top of my problems.
Interviewer	How does caring for your son affect your productivity at work?
Respondent	Sometimes you say ahaa.......... You know a human being is a human being you give in but there is where you say am I the one who is not like it`s your but it gets to much and you say let me work and I stop where I can.
Interviewer	I know that you spend much of your time caring for the patient and most of the time we struggle to make our finances stable what do you think can happen to your financial status?
Respondent	Financially when there is no you can do other things you are focused only on the patients you can not do business or do farming you are financially drained.

Case 12

Economic burden

Interviewer	How does caring for your son interfere with your source of income?
Respondent	If my husband's business lacks customers I sell my produce and I buy food or soap but when the child is sick I cannot dig this affects me.
Interviewer	As a caregiver you need money while caring for your son do you have problems with money?
Respondent	[interruption door opened] sometimes I spend little earnings to cater for the family and the patients' needs and get hard up, you may have money and used it all then sometimes my husband may work and fail to get money completely so sometimes we get stuck.
Interviewer	How does caring for your son affect your productivity at work?
Respondent	I cannot dig I have to be with him when he is fine he goes to school so I also go to the garden, when his siblings see that he has fallen at school they run and call me because the school is just near home.
Interviewer	I know that you spend much of your time caring for the patient and most of the time we struggle to make our finances stable what do you think can happen to your financial status?
Respondent	Life can become bad, I can`t buy anything like even goats or cows.

Case 13

Economic burden

Interviewer	How does caring for your son interfere with your source of income?
Respondent	Well………..when I dig I get money to buy food but when he is sick I fail to dig and my earning is affected.
Interviewer	As a caregiver you need money while caring for your son do you have problems with money?
Respondent	When he is sick I don't work and I lack money, but when I go to the garden, I harvest, keep some for our food and sell the remainder in exchange for money. .
Interviewer	How does caring for your son affect your productivity at work?
Respondent	Whenever my son is sick I lose the energy for working and I remain at hosme to look after him.
Interviewer	I know that you spend much of your time caring for the patient and most of the time we struggle to make our finances stable what do you think can happen to your financial status?
Respondent	In my heart I think that everything hurts.

Case 14

Economic burden

Interviewer	How does caring for your son interfere with your source of income?
Respondent	Well…. Before coming here I paid for her treatment I did not know about this hospital, here the drugs are for free ,expenses are not all that much except when the drugs are not available that's when we buy.
Interviewer	As a caregiver you need money while caring for your person do you has problems with money?
Respondent	Not all that
Interviewer	How does caring for your person affect your productivity at work?
Respondent	For us we don't have leave and it takes a long time to process it, if it happens that you have to attend to her if it's not time for your leave, then it 50% of your productivity is affected because we have this little money that we earn through tips as we take guides. Some other clients can also give you tips apart from salary and these other simple allowances that you get depending on how many clients you would have handled per month can be affected when you don't work but when it comes to salary it is not affected.
Interviewer	I know that you spend much of your time caring for the patient and most of the time we struggle to make our finances stable what do you think can happen to your financial status?
Respondent	Nothing much since I have no family and she is the only dependent that I am taking care of.

Case 15

Economic burden

Interviewer	How does caring for your brother interfere with your source of income?
Respondent	It is not so much because we have our brother he is assisting I just call and financially we have everything we have support.
Interviewer	As a caregiver you need money while caring for your person do you has problems with money?
Respondent	No my siblings and child help us when we get stuck.
Interviewer	How does caring for your person affect your productivity at work?
Respondent	When I go to the garden at times he escorts me he can do simple work together with me at least he also does some work.
Interviewer	I know that you spend much of your time caring for the patient and most of the time we struggle to make our finances stable what do you think can happen to your financial status?
Respondent	At times the financial stability someone is okay but you just lack that's where the problem can arise, I just can`t imagine that situation when I have failed to buy drugs for him it`s just terrible.

Case 16

Economic burden

Interviewer	How does caring for your brother interfere with your source of income?
Respondent	There is little time to do business so the earning has reduces then expenditure is also high every month I buy drugs there drugs we don`t find in the hospital they are very expensive because they are rare drugs I don`t find them in the village I have to travel to town
Interviewer	As a caregiver you need money while caring for your person do you has problems with money?
Respondent	Yes aah, the drugs are expensive and yet he cannot do without them, yet the time to look for money has been reduced to attend to him.
Interviewer	How does caring for your person affect your productivity at work?
Respondent	I have less time to attend to my business, I have lost customers and my earnings have reduced.
Interviewer	I know that you spend much of your time caring for the patient and most of the time we struggle to make our finances stable what do you think can happen to your financial status?
Respondent	Financially I am likely to lack in the long run business is likely to close and you will have no source of income.

Case 17

Economic burden

Interviewer	How does caring for your son interfere with your source of income?
Respondent	Because my earnings are seasonal and less, sometimes I lack money for feeding him and treating him.
Interviewer	As a caregiver you need money while caring for your person do you has problems with money?
Respondent	Yaah!... sometimes I lack completely because out of the little earnings I have to cater for the entire family and the patient as well.
Interviewer	How does caring for your person affect your productivity at work?
Respondent	Sometimes the attack can come early in the morning when it comes I have to stay at home, I can`t go to the garden and I can`t do any other thing for the whole day.
Interviewer	I know that you spend much of your time caring for the patient and most of the time we struggle to make our finances stable what do you think can happen to your financial status?
Respondent	Because I am the one who works If I spend all the time on the patient my family will suffer because I will miss out on the money which I would have gotten from selling tomatoes.

Case 18

Economic burden

Interviewer	How does caring for your son interfere with your source of income?
Respondent	This days I have stopped spending money on her treatment because all the drugs I have been buying for her do not cure the disease now I do not know how I can treat her she gets free treatment from the hospital.
Interviewer	As a caregiver you need money while caring for your person do you has problems with money?
Respondent	No I don`t have problems with my family because my daughter is able to pay her tuition, buy food, buy her self-drugs in addition to this the money I get from my business is enough to sustain me.
Interviewer	How does caring for your person affect your productivity at work?
Respondent	I am not affected at all, I am able to do my work well. I don`t stay with her now.
Interviewer	I know that you spend much of your time caring for the patient and most of the time we struggle to make our finances stable what do you think can happen to your financial status?
Respondent	I may end up becoming poor because i am not working but spending daily and I remain without any thing.

case 19

Economic burden

Interviewer	How does caring for your brother interfere with your source of income?
Respondent	Caring for my daughter does not interfere with my source of income because for us we dig that is how we get money for our use and food for the family.
Interviewer	As a caregiver you need money while caring for your person do you has problems with money?
Respondent	Yes I earn less yet I have patient and a family to look after.
Interviewer	How does caring for your person affect your productivity at work?
Respondent	The care giving role does not affect my family activities.
Interviewer	I know that you spend much of your time caring for the patient and most of the time we struggle to make our finances stable what do you think can happen to your financial status?
Respondent	The problem that you can face is remaining without any thing when other children fall sick you will not have money even sometimes hunger may come when that money have gone for treatment .

case 20

Economic burden

Interviewer	How does caring for your brother interfere with your source of income?
Respondent	It doesn't interfere because I am not the only one taking care of him I have siblings, my mother and some other relatives who help.
Interviewer	As a caregiver you need money while caring for your person do you has problems with money?
Respondent	Yeah..... our daddy has died so we usually rely on our relatives sometimes they don't respond like our daddy used to respond. You just have to borrow money from people for treatment.
Interviewer	How does caring for your person affect your productivity at work?
Respondent	It does not, because I am not the only person who keeps him full time.
Interviewer	I know that you spend much of your time caring for the patient and most of the time we struggle to make our finances stable what do you think can happen to your financial status?
Respondent	[interruption patient greets] yaaa.... You cannot know when the attack is coming and it can come when you don't have money. You also have other problems then you have to borrow so ideally you are affected you can lack.

case 21

Economic burden

Interviewer	How does caring for your brother interfere with your source of income?
Respondent	No it doesn't, I get money from my business and buy for her food and drinks
Interviewer	As a caregiver you need money while caring for your person do you has problems with money?
Respondent	I don't have money problems my business helps me and her father also rides a motor bicycle.
Interviewer	How does caring for your person affect your productivity at work?
Respondent	My business place is not far from home I be with her at work place, she is not seriously sick she even knows how to work she even goes to school.
Interviewer	I know that you spend much of your time caring for the patient and most of the time we struggle to make our finances stable what do you think can happen to your financial status?
Respondent	Worry money may get over

Case 22

Economic burden

Interviewer	How does caring for your brother interfere with your source of income?
Respondent	I am still a student, my parents take care of things requiring finances.
Interviewer	As a caregiver you need money while caring for your person do you has problems with money?
Respondent	There is no big problem with money towards her care, according to the treatment that she is taking it`s government that is giving an offer for that treatment so there is no big problem with caring for her with the financial status.
Interviewer	How does caring for your person affect your productivity at work?
Respondent	No, on my side it does not affect me, and if I fee on my mother's side she helps instead of having someone to help her in the hotel, they work together.
Interviewer	I know that you spend much of your time caring for the patient and most of the time we struggle to make our finances stable what do you think can happen to your financial status?
Respondent	Because now financially of course they will not be financially stable to continue helping that patient so with time they will end up living that patient just there will not be having money to buy drugs, feeds so they will end up leaving that patient.

case 23

Economic burden

Interviewer	How does caring for your brother interfere with your source of income?
Respondent	This illness is trick you can meet someone who tells you try traditional medicine, another person may tell you to go to the pastor and another person may say that this are clan things since you are desperate and confused you may try out whatever thing you are told and end up spending a lot of money without solving any problem.
Interviewer	As a caregiver you need money while caring for your person do you has problems with money?
Respondent	Mmh............yaah, there are times when he falls sick when I don`t have money, it`s worse when this happen and there are no drugs in the hospital I am forced to brow money and buy for him drugs.
Interviewer	How does caring for your person affect your productivity at work?
Respondent	It does not affect my work, the drugs he takes have helped him so much there is greet improvement seen.
Interviewer	I know that you spend much of your time caring for the patient and most of the time we struggle to make our finances stable what do you think can happen to your financial status?
Respondent	I can`t save anything, I will have many thoughts and worries and I won`t have peace at all. When you have patient you may reach somewhere and say if he is to live it`s fine or if he dies it`s God to decide.

Case 24

Economic burden

Interviewer	How does caring for your brother interfere with your source of income?
Respondent	Most of the money I get I spend on her treatment, transport and feeding but when she is sick that means I suspend my garden work and my earning is affected.
Interviewer	As a caregiver you need money while caring for your person do you has problems with money?
Respondent	Yes being a peasant farmer I earn less, the money I get I buy food, educate her siblings and my other grand children whose parents died surely I lack money most of the time.
Interviewer	How does caring for your person affect your productivity at work?
Respondent	I am not affected by caring for her, I go with her in my garden if she refuses to go along with me to the garden I leave her to stay at home with other children.
Interviewer	I know that you spend much of your time caring for the patient and most of the time we struggle to make our finances stable what do you think can happen to your financial status?
Respondent	The disease progresses when you lack money to buy drugs you may end up remaining poor and lacking.

Case 25

Economic burden

Interviewer	How does caring for your brother interfere with your source of income?
Respondent	There is less time to attend to business, no hope for employment opportunity due to stigma. At times when I get a job I end up losing it because of my brother's sickness I absentee myself from work to look after him my income is affected like that.
Interviewer	As a caregiver you need money while caring for your person do you has problems with money?
Respondent	There is a problem when am caring for the patient I cannot have another time to look for money and even if I am to look for it any money that I get it will automatically being transferred for caring this patient and that is a big challenge to me.
Interviewer	How does caring for your person affect your productivity at work?
Respondent	There is negative productivity because all the time you think about the patient so I can not perform according to the organization expectations and objectives.
Interviewer	I know that you spend much of your time caring for the patient and most of the time we struggle to make our finances stable what do you think can happen to your financial status?
Respondent	I become broke all the time because whichever money I get goes for treatment you cannot have money when you have a patient because you lack time to work and become financially stable.

case 26

Economic burden

Interviewer	How does caring for your daughter interfere with your source of income?
Respondent	My earnings are less the little money I get I buy food and use for treating her.
Interviewer	As a caregiver you need money while caring for your person do you has problems with money?
Respondent	I face challenges of buying her food and drugs but because I don't want her to think a lot I try to provide her with everything that she needs.
Interviewer	How does caring for your person affect your productivity at work?
Respondent	I work but all my minds be on the sick child whom I have left at home I keep on wondering whether I will find my child alive or dead.
Interviewer	I know that you spend much of your time caring for the patient and most of the time we struggle to make our finances stable what do you think can happen to your financial status?
Respondent	Because I want my child to be fine, many times I borrow money which I pay back with interest that has made me to become poor but what is the use of keeping money when your child is dying.

case. 27

Economic burden

Interviewer	How does caring for your brother interfere with your source of income?
Respondent	I have spent a lot of money on treatment, I have to go to the hospital which is a bit far, I have to buy for him food and give him a lot of drinks.
Interviewer	As a caregiver you need money while caring for your person do you has problems with money?
Respondent	Yea when I don't have money I have to think of how I will get the money.
Interviewer	How does caring for your person affect your productivity at work?
Respondent	I work when my minds are there at home when I am on duty I start thinking whether the person I have asked to help me is still there or not.
Interviewer	I know that you spend much of your time caring for the patient and most of the time we struggle to make our finances stable what do you think can happen to your financial status?
Respondent	It disturbs a lot because the money that you have has gotten can finished.

case 28

Economic burden

Interviewer	How does caring for your brother interfere with your source of income?
Respondent	Yes caring for her limits me from doing my garden work most of the time.
Interviewer	As a caregiver you need money while caring for your person do you has problems with money?
Respondent	since I am a peasant I am not somebody who is getting daily money, my money comes seasonally, I normally get some hardship to treat her because her medication needs money and I don`t have enough money this forces me not to follow the right procedure of medication.
Interviewer	How does caring for your person affect your productivity at work?
Respondent	Sometimes I don`t go to the garden I remain at home taking care of her.
Interviewer	I know that you spend much of your time caring for the patient and most of the time we struggle to make our finances stable what do you think can happen to your financial status?
Respondent	Financially I am not stable because most of the time I have to give her care.

Case 29

Economic burden

Interviewer	How does caring for your daughter interfere with your source of income?
Respondent	Yes caring for her limits me from doing my garden work most of the time my business have all closed because I tried employing people but they could mismanage my funds.
Interviewer	As a caregiver you need money while caring for your person do you has problems with money?
Respondent	I am not somebody who is getting daily money, I normally get some hardship to treat her because her medication needs money and I don`t have enough money this forces me not to follow the right procedure of medication.
Interviewer	How does caring for your person affect your productivity at work?
Respondent	Sometimes I don`t go to the garden I remain at home to take care of her.
Interviewer	I know that you spend much of your time caring for the patient and most of the time we struggle to make our finances stable what do you think can happen to your financial status?
Respondent	Financially I will not become stable and life can be very hard you know………the life of today everything needs money.

case 30

Economic burden

Interviewer	How does caring for your brother interfere with your source of income?
Respondent	It interferes with my income because the only one taking care of her and I have no stable source of income. No one can employ you and entertain you being absent all the time in the name of having a sick child.
Interviewer	As a caregiver you need money while caring for your person do you have problems with money?
Respondent	Yeah...... like I said I am the sole bread winner and I am unemployed. I only rely on agriculture which is tricky sometimes you get at times you don't you only incur losses.
Interviewer	How does caring for your person affect your productivity at work?
Respondent	It does partially, because the patient takes 50% of my time and I spend the other half for doing my work I don't give my best my work and my family suffers the consequence.
Interviewer	I know that you spend much of your time caring for the patient and most of the time we struggle to make our finances stable what do you think can happen to your financial status when you spend all your time looking after the patient?
Respondent	[interruption patient greets] yaaa.... I will automatically become poor, my family will lead a miserable life and the patient may end up dying before her appointed time becase I will fail to cater for her I mean everything needs money.
