# Supplementary material for: Lived experiences of caregivers of persons with epilepsy attending an epilepsy clinic at a tertiary hospital, eastern Uganda: A phenomenological approach
Source: PLoS One. 2023 Jul 18;18(7):e0274373. doi: 10.1371/journal.pone.0274373 (PMC10353802; doi:10.1371/journal.pone.0274373)
Supplement: S1 Data — (ZIP) [file pone.0274373.s001.zip › Psychological Burden.pdf]

Case 1

SECTION B: PSYCHOLOGICAL BURDEN

**Interviewer**	How does caring for a person with epilepsy influence your thoughts about day to day living?
**Respondent:**	*I think perhaps she may not get cured that makes me worry. I feed her on porridge and sometimes I lack money for buying milk and porridge flour...... I get worried about how she will feed.....!*
**Interviewer**	Ok! How does caring for a person with epilepsy interfere with your personal relationship with others?
**Respondent**	*They undermine me and they laugh at me that I produced a lame child*
**Interviewer**	*Hmmm.... Do you have time for yourself, can you elaborate?*
**Respondent**	*Yes, I can dig and do any thing*
**Interviewer**	*How do you feel when separated from the person you are caring for?*
**Respondent**	*Yah.... I can't be where she is not, even the Dad says I take her to his home but I cannot allow.*
**Interviewer**	*How does caring for a person with epilepsy influence your behavior?*
**Respondent**	*[Interruption .....child coughs] I don't bark at her , I give her toys to play with ,I also relate well with others*

Case 2

SECTION B: PSYCHOLOGICAL BURDEN

**Interviewer**	How does caring for a person with epilepsy influence your thoughts about day to day living?
**Respondent:**	*As for me when she falls , I feel bad, the last episode which happened recently she fell the whole day and stayed hungry the whole day she didn't eat.mmh……. if she is fine she can dig or even fetch water. In my thoughts I think it will be one day it does like that and she dies.*
**Interviewer**	How does caring for a person with epilepsy interfere with your personal relationship with others?
**Respondent**	*Some people fear her they say that Ahaa….. this child falls down we don't want her to be with our children because she will infect them some tell me to isolate her to eat alone, to get for her a basin to use alone and get for her a personal cup as well but I talk well with those people.*
**Interviewer**	*Do you have time for yourself, can you elaborate?*
**Respondent**	*I have enough time but when I go somewhere I make sure that I come back quickly because I know anytime she can get the attack.*
**Interviewer**	*How do you feel when separated from the person you are caring for?*
**Respondent**	*I fear because when she is alone and she goes to cook she might fall in fire in fact when I go somewhere I can't sleep there I tell them that I have left a patient at home.*
**Interviewer**	*How does caring for a person with epilepsy influence your behavior?*
**Respondent**	*I get shocked when she has fallen, but when she is fine we stay well and do everything together happily.*

Case 3

SECTION B: PSYCHOLOGICAL BURDEN

**Interviewer**	How does caring for your daughter influence the way you think about life?
**Respondent:**	My daughter is improving she has not been walking but from the time I began treatment she has started crawling and with time I will take her to school.[mother laughs.....]
**Interviewer**	How does caring for a person with epilepsy interfere with your personal relationship with others?
**Respondent**	*My friends tell me to take to take my child so that their kids can carry her.*
**Interviewer**	*Do you have time for yourself, can you elaborate?*
**Respondent**	*[door opened] Yes because I attend to my business and my daughter in fact I rather pay attention to my daughter than my business I normally don't want her to cry and I don't want to leave her under the care of another person.*
**Interviewer**	*How do you feel when separated from your daughter?*
**Respondent**	*I feel very bad and have very bad thoughts I don't like leaving her and when I go away she remains crying throughout the day.*
**Interviewer**	*How does caring for a person with epilepsy influence your behavior?*
**Respondent**	*I behave well my behavior doesn't change, … … .....i am free.*

Case 4

SECTION B: PSYCHOLOGICAL BURDEN

**Interviewer**	How does caring for your son influence the way you think about life?
**Respondent:**	Because I know that God helps I don't worry, those years back my son was badly off but now he has improved, he is in a good condition I can tell him to bring a plate and I serve him food and he does.
**Interviewer**	How does caring for a person with epilepsy interfere with your personal relationship with others?
**Respondent**	*Some people fear me and they isolate themselves from me.some of them fear my son and think that he will infect their children nowadays i restrict him from moving to the neighbors' homes we stay in our home .*
**Interviewer**	*Do you have time for yourself, can you elaborate?*
**Respondent**	*The time I have is enough. My elder son is married sometimes I leave him with my daughter in law then I go and do my other things or I lock him inside the house because I can't leave him outside but I don't take long when I go to the garden I make sure that I come back quickly.*
**Interviewer**	*How do you feel when separated from your daughter?*
**Respondent**	*Yah……...Nurse I feel bad [the mother laughs]when I go somewhere immediately I arrive I tell them I tell them I have left a sick child behind since they understand me and the child's condition they allow me to go back and attend to him.*
**Interviewer**	*How does caring for a person with epilepsy influence your behavior?*
**Respondent**	*I talk to everyone well irrespective of their age; in fact people say that I am a very jolly and patient woman because it's not easy to keep a child with such a condition.*

Case 5

SECTION B: PSYCHOLOGICAL BURDEN

**Interviewer**	How does caring for your son influence the way you think about life?
**Respondent:**	At first I used to worry but now I have left everything to God even though I worry there is nothing to do life is not easy because I am not employed , money is not there……… educating children is difficult even feeding is not easy .
**Interviewer**	How does caring for a person with epilepsy interfere with your personal relationship with others?
**Respondent**	*It can't affect me because we are free we have no problem in relating.*
**Interviewer**	*Do you have time for yourself, can you elaborate?*
**Respondent**	*Mmmh…..I feel that the time I have is not enough because I have to go to the garden every day after digging I have to look for food and prepare for the family and I also have to take care of the patient.*
**Interviewer**	*How do you feel when separated from your daughter?*
**Respondent**	*Okay……… when I go somewhere all my minds be behind because I keep on thinking and asking myself is he really well or not*
**Interviewer**	*How does caring for a person with epilepsy influence your behavior?*
**Respondent**	*Mmmh………sometimes I feel annoyed , I usually get tired and when he improves I tend to think that he is going to be well only to see the episode re- occurring again.*

Case 6

SECTION B: PSYCHOLOGICAL BURDEN

**Interviewer**	How does caring for your husband influence the way you think about life?
**Respondent:**	At first I used to worry but right now I have left everything to God even though I worry, there is nothing to do. Life is not easy I am not employed; money is not there to educate children even feeding them is not easy.
**Interviewer**	How does caring for a person with epilepsy interfere with your personal relationship with others?
**Respondent**	*I have no problem in relating with people, I am free my husband's sickness does not affect my relationship with others.*
**Interviewer**	*Do you have time for yourself, can you elaborate?*
**Respondent**	*The time I have is not enough because I have to dig every day when I come back from the garden I look for food to prepare and I still have to care of my sick husband.*
**Interviewer**	*How do you feel when separated from your grandson?*
**Respondent**	*Ok……. When I go somewhere, all my minds be behind I keep on thinking whether my husband is fine or not.*
**Interviewer**	*How does caring for a person with epilepsy influence your behavior?*
**Respondent**	*Well at times i feel annoyed because I get tired sometimes he becomes fine and I tend to think that he is getting well then the episode re-occurs again.*

Case 7

SECTION B: PSYCHOLOGICAL BURDEN

**Interviewer**	How does caring for your Daughter influence the way you think about life?
**Respondent:**	I worry a lot that this child will die any time. I think a lot because all the time she is sick even right now we are hospitalized but when we are discharged I know she is going to get an attack any time that makes me worry. My husband used to help me but when he realized that this disease cannot be cured he does not give me any help. When I give him a call, he switches off his phone now I am the mother as well as the father to this child that disturbs me. This sickness I wish God would heal my daughter.
**Interviewer**	How does caring for a person with epilepsy interfere with your personal relationship with others?
**Respondent**	*[Interruption people talking outside] people look at us and talk ill about us where we are renting people don't want their children to be with this one. They talk to me that we be there like that for us we are saved and we pray for them*
**Interviewer**	*Do you have time for yourself, can you elaborate?*
**Respondent**	*The time I have is not enough when she falls sick I become energyless and I loose hope. I don't have time to even go to the village my siblings don't visit me because of her illness and I cant get some one to help me keep her.*
**Interviewer**	*How do you feel when separated from your daughter?*
**Respondent**	*When I am away from her I am not settled ,It affects me and even the child herself doesn't feel happy.When it gets dark before I get back home no one allows her to enter in their house, when I get worried because I may find when she has gone to roam aimlessly if she does not see me yet the attack can come at any time.*
**Interviewer**	*How does caring for a person with epilepsy influence your behavior?*
**Respondent**	*Me I am free I don't have anything to think about whether someone likes me or not I don't mind. I can give them food and they refuse to eat. Some of them don't want my daughter hold there children.*

Case 8

SECTION B: PSYCHOLOGICAL BURDEN

**Interviewer**	How does caring for your Daughter influence the way you think about life?
**Respondent:**	I worry all the time, I am thinking will my child get well or she will die. I am just wondering which hospital manages this condition, sometimes you may wonder are this worldly because people tell you to try out many things, you may get confused and wonder whether prayers work.
**Interviewer**	How does caring for a person with epilepsy interfere with your personal relationship with others?
**Respondent**	*People don`t like me they isolate themselves from me because they say that the disease is contagious they don`t want to be near me .*
**Interviewer**	*Do you have time for yourself, can you elaborate?*
**Respondent**	*I don`t have enough time because all the time I am worried, this is a serious problem, at night I don`t sleep soundly because I have to keep monitoring how she is, I don`t mind about myself because much of my time and attention is taken by the patient.*
**Interviewer**	*How do you feel when separated from your daughter?*
**Respondent**	*I feel a lot of pain, because your child who came out of your womb how can you refuse to get near her I feel pity all the time.*
**Interviewer**	*How does caring for a person with epilepsy influence your behavior?*
**Respondent**	*If she is sick I treat her like a baby because if I shout at her and beat her she becomes weak. I don`t abuse her all the time I take care of her, I bathe her, I look for food, I organize where she sleeps and make sure that she is happy.*

Case 9

SECTION B: PSYCHOLOGICAL BURDEN

**Interviewer**	How does caring for your Daughter influence the way you think about life?
**Respondent:**	I worry I want her to be cured I went to the hospital and they gave her medicine but they are not enough.
**Interviewer**	How does caring for a person with epilepsy interfere with your personal relationship with others?
**Respondent**	*We be with people but she doesn`t be near them people fear they say that she gets epileptic attacks and they fear that they may get infected.*
**Interviewer**	*Do you have time for yourself, can you elaborate?*
**Respondent**	*Yes I have enough time, sometimes I visit my relatives but in my heart I pray that God keeps her.*
**Interviewer**	*How do you feel when separated from your daughter?*
**Respondent**	*I felt so bad, right now that we are hospitalized here my thoughts are on her the father came here in the morning and I asked him how is that child now that I am not at home and he told me she is their like that.*
**Interviewer**	*How does caring for a person with epilepsy influence your behavior?*
**Respondent**	*There is no way caring for her influences may behave I give her medicine whatever food we eat she also eats because they are all my children we don`t segregate her.*

Case 10

SECTION B: PSYCHOLOGICAL BURDEN

**Interviewer**	How does caring for your Son influence the way you think about life?
**Respondent:**	I have hope that God will heal him.
**Interviewer**	How does caring for a person with epilepsy interfere with your personal relationship with others?
**Respondent**	*They help me, they don`t fear, there is no problem.*
**Interviewer**	*Do you have time for yourself, can you elaborate?*
**Respondent**	*I have less time, I help the patient by taking him, I help him bathe, I cook food for him, I be near him all the time so that he does not go any where*
**Interviewer**	*How do you feel when separated from your daughter?*
**Respondent**	*I feel bad because if I am not there there is no one who can take care of him like I do ,though they look after him they don`t take care of him like myself so when I am away from home I worry a lot but I leave everything into God.*
**Interviewer**	*How does caring for a person with epilepsy influence your behavior?*
**Respondent**	*The patient disturbs it makes my behavior change but I be strong, I take got care of him whatever he says I answer him well, I don`t treat him badly because I know that he is sick.*

Case 11

SECTION B: PSYCHOLOGICAL BURDEN

**Interviewer**	How does caring for your person afflicted with epilepsy influence the way you think about life?
**Respondent:**	It is not easy because sometimes she becomes rebellious she can refuse those drugs you try to hustle your time and stress yourself it appears as if your forcing someone to love something which helps her and yet she needs it.
**Interviewer**	How does caring for a person with epilepsy interfere with your personal relationship with others?
**Respondent**	*It occupies me because it takes my time if I go away and this person falls down and may it is on fire or on water when I think about this I can`t move even if I leave somebody behind my mind is always thinking about the patient.*
**Interviewer**	*Do you have time for yourself, can you elaborate?*
**Respondent**	*Sometimes it is hard if you are two it is ok but if your alone you cannot even be in your home because your responsible in taking care of the patient you cannot be with your family when you are supposed to be with them*
**Interviewer**	*How do you feel when separated from your daughter?*
**Respondent**	*I feel bad because if I am not there there is no one who can take care of him like I do ,though they look after him they don`t take care of him like myself so when I am away from home I worry a lot but I leave everything into God.*
**Interviewer**	*How does caring for a person with epilepsy influence your behavior?*
**Respondent**	*The patient disturbs it makes my behavior change but I be strong, I take got care of him whatever he says I answer him well, I don`t treat him badly because I know that he is sick.*

Case 12

SECTION B: PSYCHOLOGICAL BURDEN

**Interviewer**	How does caring for your person afflicted with epilepsy influence the way you think about life?
**Respondent:**	I don`t know whether she will be fine or how will she be in future.
**Interviewer**	How does caring for a person with epilepsy interfere with your personal relationship with others?
**Respondent**	*When she gets an attack they fear her, but I also restrict the child from moving to peoples homes.*
**Interviewer**	*Do you have time for yourself, can you elaborate?*
**Respondent**	*Yes there is enough time for self-care and doing other personal activities.*
**Interviewer**	*How do you feel when separated from your daughter?*
**Respondent**	*Eeh!there I feel bad no one else can take care of her like I do.*
**Interviewer**	*How does caring for a person with epilepsy influence your behavior?*
**Respondent**	*I am okey I only get harsh when I stop her from doing something and she disobey*

Case 13

SECTION B: PSYCHOLOGICAL BURDEN

**Interviewer**	How does caring for your person afflicted with epilepsy influence the way you think about life?
**Respondent:**	[nods head] I think that God may heal him.
**Interviewer**	How does caring for a person with epilepsy interfere with your personal relationship with others?
**Respondent**	*Whenever he moves or goes to the neighbors place he fits all time I don't want him to go anywhere I am with him all the time. The friends beat him because he likes fighting a lot but my neighbors treat me well.*
**Interviewer**	*Do you have time for yourself, can you elaborate?*
**Respondent**	*I don't do anything all the time he disturbs and yet I have a young baby and their father is not near he works far from home and can spend a long time before coming home.*
**Interviewer**	*How do you feel when separated from your son?*
**Respondent**	*It affects me I feel bad because I didn't want him to be the way he is. You know…… in our hearts we are not the same there are good and bad people, I fear if I leave him behind he may be mistreated.*
**Interviewer**	*How does caring for a person with epilepsy influence your behavior?*
**Respondent**	*It take good care of him because when I beat him he doesn't understand ,even others I don't get annoyed with them because this child does things when he doesn't know.*

Case 14

SECTION B: PSYCHOLOGICAL BURDEN

**Interviewer**	How does caring for your person afflicted with epilepsy influence the way you think about life?
**Respondent:**	Taking care of her wouldn't be a problem it only troubles me if she is attacked whenever she is attacked she becomes very weak and at times I even fail to know what to do because at times she is attacked immediately after taking the drugs
**Interviewer**	How does caring for a person with epilepsy interfere with your personal relationship with others?
**Respondent**	*It has not interfered all that because the way I take her, I take her just like my sister and whenever she is attacked I handle it privately I don't leave her to go to lonely places but the attack comes when she is at home so it is like most people don't even know that she is sick.*
**Interviewer**	*Do you have time for yourself, can you elaborate?*
**Respondent**	*When it comes to doing my other things I feel that I have enough time.*
**Interviewer**	*How do you feel when separated from your person?*
**Respondent**	*For sure when I am separated from her I really feel uncomfortable because one thing I know is that the parents cannot cater for her because the situation is just not all that good ,but whenever I am with her I feel that everything is okay like I told you I take her as my sister.*
**Interviewer**	*How does caring for a person with epilepsy influence your behavior?*
**Respondent**	*Obviously me I just feel happy because that is what I am supposed to do in accordance to what the bible tells us we are supposed to cater for those who suffer.*

case 15

SECTION B: PSYCHOLOGICAL BURDEN

**Interviewer**	How does caring for your person afflicted with epilepsy influence the way you think about life?
**Respondent:**	You have to put in the time to be close to him put aside money every month getting drugs because there are some drugs that are not available then you ……………to hospital from Palisa district. You have to think even when you are not with him and you go somewhere you cannot leave him alone someone has to be weak because of the attacks.
**Interviewer**	How does caring for a person with epilepsy interfere with your personal relationship with others?
**Respondent**	*It takes some of the time, but I have not seen any problem at least family and close relatives not most of the people knows he is sick we relate well.*
**Interviewer**	*Do you have time for yourself, can you elaborate?*
**Respondent**	*It is not enough especially when before we brought him to start on medication he used to get frequent attacks therefore it called for us to be close and at times you have to be close business in case the attack comes it`s a problem because you know as sometimes mum may have to travel and attend burials but you have to forego business and attend to him.*
**Interviewer**	*How do you feel when separated from your person?*
**Respondent**	*There is that worry because you imagine may be it might come seriously when the old woman may not handle, you don`t feel comfortable wherever you go.*
**Interviewer**	*How does caring for a person with epilepsy influence your behavior?*
**Respondent**	*.for me normally my mood changes when he becomes resistant to what I feel he should do, I get annoyed with him because for instance they are instances he is supposed to come for renew and he seem to have given up he says for me at my age even if anything happens just leave me sometimes you may have to wish to use force to bring him to the hospital but again I understand the repercussions.*

Case 16

SECTION B: PSYCHOLOGICAL BURDEN

**Interviewer**	How does caring for your person afflicted with epilepsy influence the way you think about life?
**Respondent:**	You have to put in the time to be close to him put aside money every month getting drugs because there are some drugs that are not available then you ……………to hospital from Palisa district. You have to think even when you are not with him and you go somewhere you cannot leave him alone someone has to be weak because of the attacks.
**Interviewer**	How does caring for a person with epilepsy interfere with your personal relationship with others?
**Respondent**	*It takes some of the time, but I have not seen any problem at least family and close relatives not most of the people knows he is sick we relate well.*
**Interviewer**	*Do you have time for yourself, can you elaborate?*
**Respondent**	*It is not enough especially when before we brought him to start on medication he used to get frequent attacks therefore it called for us to be close and at times you have to be close business in case the attack comes it`s a problem because you know as sometimes mum may have to travel and attend burials but you have to forego business and attend to him.*
**Interviewer**	*How do you feel when separated from your person?*
**Respondent**	*There is that worry because you imagine may be it might come seriously when the old woman may not handle, you don`t feel comfortable wherever you go.*
**Interviewer**	*How does caring for a person with epilepsy influence your behavior?*
**Respondent**	*.for me normally my mood changes when he becomes resistant to what I feel he should do, I get annoyed with him because for instance they are instances he is supposed to come for renew and he seem to have given up he says for me at my age even if anything happens just leave me sometimes you may have to wish to use force to bring him to the hospital but again I understand the repercussions.*

case 1 ₮

SECTION B: PSYCHOLOGICAL BURDEN

**Interviewer**	How does caring for your person afflicted with epilepsy influence the way you think about life?
**Respondent:**	I normally think of how to treat my son I do not have money to look after the patient well, I grow tomatoes and sell to sustain my family, sometimes if the season is not good my harvest is affected and this makes me worried because I cannot afford his treatment
**Interviewer**	How does caring for a person with epilepsy interfere with your personal relationship with others?
**Respondent**	*we talk well, there are some days when he is attacked and I am not around but my friends and neighbors who like me come and help*
**Interviewer**	*Do you have time for yourself, can you elaborate?*
**Respondent**	*The time is less because I have to take care of him and when I go to the garden I don`t spend their long.*
**Interviewer**	*How do you feel when separated from your person?*
**Respondent**	*I think about him when I have moved away from home to sell tomatoes but I leave him upon God`s hands.*
**Interviewer**	*How does caring for a person with epilepsy influence your behavior?*
**Respondent**	*If i am with people some thoughts go off and I become okay but sometimes I get affected when I am alone and thinking a lot.*

Case 18

SECTION B: PSYCHOLOGICAL BURDEN

**Interviewer**	How does caring for your daughter influence the way you think about life?
**Respondent:**	Me I just want treatment, talk of treatment only because I know it can make her to become fine.
**Interviewer**	How does caring for a person with epilepsy interfere with your personal relationship with others?
**Respondent**	*Caring for my daughter does not interfere with the way I relate with people because when you see my daughter she is fat and health you cannot know that she has this disease in fact most people do not know that she is sick.*
**Interviewer**	*Do you have time for yourself, can you elaborate?*
**Respondent**	*Yes the time I have is enough for me because I only have two grown up daughters who are g in higher institutions of learning they only come home briefly for holidays so I am able to do my work of tailoring well.*
**Interviewer**	*How do you feel when separated from your person?*
**Respondent**	*I am only scared and I feel very bad because that she is not with me but being an adult I can not keep her at home most of the times she only tells me mum I got an attack it`s an a shaming disease but there is nothing to do.*
**Interviewer**	*How does caring for a person with epilepsy influence your behavior?*
**Respondent**	*My behavior have not changed at all since diagnosed of this disease I am a jolly person and I relate well with people .*

Case 17

SECTION B: PSYCHOLOGICAL BURDEN

**Interviewer**	How does caring for your person afflicted with epilepsy influence the way you think about life?
**Respondent:**	These days he is improving he is not like he used to be before we started treatment I think with time he will become fine.
**Interviewer**	How does caring for a person with epilepsy interfere with your personal relationship with others?
**Respondent**	*I relate well with my neighbuors we have never had problem since we began staying to gather.*
**Interviewer**	*Do you have time for yourself, can you elaborate?*
**Respondent**	*I have enough time for my self because I am able to do what I like to do.*
**Interviewer**	*How do you feel when separated from your person?*
**Respondent**	*I don`t feel bad if there is treatment because I know that even though I go far she will be using the drugs, but if there are no drugs I feel bad because when the attack gets her it can treat her in a bad way.*
**Interviewer**	*How does caring for a person with epilepsy influence your behavior?*
**Respondent**	*I behave well, I don`t get annoyed of her, generally my behavior is not bad.*

Case 20

SECTION B: PSYCHOLOGICAL BURDEN

**Interviewer**	How does caring for your person afflicted with epilepsy influence the way you think about life?
**Respondent:**	Sometimes I feel pity feel a shamed, sometimes I can feel as if I am defeated as if he will never respond because he takes drugs but again still experiences those attacks, sometimes I wonder whether these are demons or a natural disease I just feel I am confused.
**Interviewer**	How does caring for a person with epilepsy interfere with your personal relationship with others?
**Respondent**	*I relate well with others caring for my brother does not interfere with my relationship with people.*
**Interviewer**	*Do you have time for yourself, can you elaborate?*
**Respondent**	*The time I have is enough only when he gets the attack that is when I have to suspend other activities.*
**Interviewer**	*How do you feel when separated from your person?*
**Respondent**	*I feel bad, because I don`t know what may happen, to him, I don`t where he will get the attack from it may be near the road I get so worried because I don`t know what may happen.*
**Interviewer**	*How does caring for a person with epilepsy influence your behavior?*
**Respondent**	*I have now come to understand his condition I cannot run away from him I put myself in his shoes and try to imagine If I was the one I wouldn`t wish to be segregated.*

Case 21

SECTION B: PSYCHOLOGICAL BURDEN

**Interviewer**	How does caring for your person afflicted with epilepsy influence the way you think about life?
**Respondent:**	I think she may get well
**Interviewer**	How does caring for a person with epilepsy interfere with your personal relationship with others?
**Respondent**	*They laugh at me when that disease comes they don't want to get close to me. They say that one falls down don't go near her you will be infected.*
**Interviewer**	*Do you have time for yourself, can you elaborate?*
**Respondent**	*.yes, I have enough time for self care.*
**Interviewer**	*How do you feel when separated from your person?*
**Respondent**	*I worry she might fall in fire.*
**Interviewer**	*How does caring for a person with epilepsy influence your behavior?*
**Respondent**	*I don't bather , I don't shout at her, I treat her well and feeed her on time*

Case 22

SECTION B: PSYCHOLOGICAL BURDEN

**Interviewer**	How does caring for your person afflicted with epilepsy influence the way you think about life?
**Respondent:**	There is a way it has affected her and her expectations because was also studying but because of that sickness she was not able to achieve what she expected she failed even to complete P.7 because of that sickness it comes even when she is in class. She used not to collapse but she could change her minds, she used to like fighting a lot she could not listen to teachers so it affected her, her future expectations and up to now you talk to her she has no minds of going back to school she likes staying alone.
**Interviewer**	How does caring for a person with epilepsy interfere with your personal relationship with others?
**Respondent**	*No, it doesn't interfere, most of the time, she is in mums hotel, she can do her well and for me my work is just to get for her drugs.*
**Interviewer**	*Do you have time for yourself, can you elaborate?*
**Respondent**	*.yes I only pick dress for her from te hospital.*
**Interviewer**	*How do you feel when separated from your person?*
**Respondent**	*.i feel bad because people don`t like her because her behavior, but since I understand her condition it`s not a permanent condition, I like her because it`s just a short time condition.*
**Interviewer**	*How does caring for a person with epilepsy influence your behavior?*
**Respondent**	*.sometimes as for me my Mzee has a step mum and I have my brothers and sisters if it happens they end up taking something's sometimes it can annoy me if it happens that they over talk but I don`t talk to her I talk to them.*

Case 23

SECTION B: PSYCHOLOGICAL BURDEN

**Interviewer**	How does caring for your person afflicted with epilepsy influence the way you think about life?
**Respondent:**	What pains me most is that he is sick, in my thoughts I pray that God may heal him, I always think this child I had hoped that he would help me in future but now he is sick in my heart I feel pain.
**Interviewer**	How does caring for a person with epilepsy interfere with your personal relationship with others?
**Respondent**	*Not as such, whenever he goes to play and he gets an attack my neighbours call me, we relate well with them when if falls somewhere my neighbours call me tell me my child is here.*
**Interviewer**	*Do you have time for yourself, can you elaborate?*
**Respondent**	*There is enough time because he is not a worrying patient, he is not badly enough, I can move to my village go and dig, do my business.*
**Interviewer**	*How do you feel when separated from your person?*
**Respondent**	*I feel bad when I think about him but if I am doing other things and I am not thinking about him I feel comfortable. Whenever I am away I call back home and find out how he is, you know whenever you have a patient with a chronic illness you reach somewhere and you get used to it.*
**Interviewer**	*How does caring for a person with epilepsy influence your behavior?*
**Respondent**	*When I used to be at home I used to think it`s me with such a patient alone but when I came to the hospital I saw my son is better off because I saw other children whose conditions were worse than mine that made me to become stronger.*

case 24

SECTION B: PSYCHOLOGICAL BURDEN

**Interviewer**	How does caring for your person afflicted with epilepsy influence the way you think about life?
**Respondent:**	I think of bringing her here so that she gets treatment
**Interviewer**	How does caring for a person with epilepsy interfere with your personal relationship with others?
**Respondent**	*Caring for my daughter does not interfere with the way I relate with others.*
**Interviewer**	*Do you have time for yourself, can you elaborate?*
**Respondent**	*Though my daughter is sick she does not disturb me, she only eats and sleeps there after I feel I have enough time for myself.*
**Interviewer**	*How do you feel when separated from your person?*
**Respondent**	*When I am away from home I don`t get worried because this drugs have helped her.*
**Interviewer**	*How does caring for a person with epilepsy influence your behavior?*
**Respondent**	*At times she annoys me because of her illness but I behave well since I understand that she is sick.*

Case 25

SECTION B: PSYCHOLOGICAL BURDEN

**Interviewer**	How does caring for your person afflicted with epilepsy influence the way you think about life?
**Respondent:**	Sometimes I think that with time he will be well, at times I think that he was born unlucky some where some how because having such a kind of disease is like someone who doesn`t look normal among other people so I think he is unlucky.
**Interviewer**	How does caring for a person with epilepsy interfere with your personal relationship with others?
**Respondent**	*Sometimes I feel a shamed before my friends by thinking that may be I am a victim as well and there fore it cuts off some relationship with my friends*
**Interviewer**	*Do you have time for yourself, can you elaborate?*
**Respondent**	*Really no, I don't have enough time because I have some other things that I would like to do but because of caring for that patient I feel like I don't have enough time . you know with business when you are not there you end up making loses. It can lead to the collapse of the business.*
**Interviewer**	*How do you feel when separated from your person?*
**Respondent**	*I somehow feel peace of mind in me but when I thank about him being a brother I end up having trouble within me because your own brother leaving him just there because of sickness it doesn't reflect in you to be a caring person.*
**Interviewer**	*How does caring for a person with epilepsy influence your behavior?*
**Respondent**	*My behavior is influenced negatively I feel out casted, I become humble and ready for any action that may occur on him physically. I am humbled because I cannot express my self in public some people may end up talking to me badly and every one may think that I have this disease as well. You know people funny comments.*

case 26

SECTION B: PSYCHOLOGICAL BURDEN

**Interviewer**	How does caring for your person afflicted with epilepsy influence the way you think about life?
**Respondent:**	I think about the child and I wonder whether she will get fine. When I take her to the hospital I am discharged and the health workers tell me that this disease has no cure.
**Interviewer**	How does caring for a person with epilepsy interfere with your personal relationship with others?
**Respondent**	*.my neighbours treat me in a bad way they don't want my child to go and play with their children in their homes, they say that he may infect them.*
**Interviewer**	*Do you have time for yourself, can you elaborate?*
**Respondent**	*I don't have enough time for myself I leave her with her mother and go for the work, if the mother is going to the garden, she locks her in the house there is no one to remain with her at home.*
**Interviewer**	*How do you feel when separated from your person?*
**Respondent**	*I feel so bad that sometimes my child may fall and die I ask myself what can I do, where do this disease come from but I get no answer.*
**Interviewer**	*How does caring for a person with epilepsy influence your behavior?*
**Respondent**	*.i think a lot about her illness I don't know where it came from and people tell me to try visiting many places so that she can get healed..*

Case 27

SECTION B: PSYCHOLOGICAL BURDEN

**Interviewer**	How does caring for your person afflicted with epilepsy influence the way you think about life?
**Respondent:**	In fact I have tried taking the child to different hospitals for treatment but there is no improvement that I have seen the attacks still come I was told of another hospital I want to try them if things fail I will resort to using the local herbs.
**Interviewer**	How does caring for a person with epilepsy interfere with your personal relationship with others?
**Respondent**	*In fact other people are not happy with the sickness of the child and they don't want to get near him.*
**Interviewer**	*Do you have time for yourself, can you elaborate?*
**Respondent**	*No I don't have enough time because I cannot sit with him for the whole day at home and yet i have to look for what they will eat*
**Interviewer**	*How do you feel when separated from your person?*
**Respondent**	*I don't always feel okay I always feel it when he is left alone at home minus the mother, I don't feel happy I have to work and make up time rush back home and check on him.*
**Interviewer**	*How does caring for a person with epilepsy influence your behavior?*
**Respondent**	*I am not always happy I am pre-occupied with thoughts.*

case 2 B

SECTION B: PSYCHOLOGICAL BURDEN

**Interviewer**	How does caring for your sister influence the way you think about life?
**Respondent:**	The way she is suffering on my side I am worried because when the sickness comes it attacks her terribly and she suffers a lot also I am a peasant farmer, our parents passed away now I am the only one who is supposed to look after her treating her becomes hard at times when because it is hard for me to get money.
**Interviewer**	How does caring for a person with epilepsy interfere with your personal relationship with others?
**Respondent**	*Yea.....people around us in our village fear to come near her because they think that when they come near her this sickness can easily be transmitted to them I get hard time because people fear to come and help me so I suffer alone.*
**Interviewer**	*Do you have time for yourself, can you elaborate?*
**Respondent**	*I don't have enough time for myself because I fear sometimes to leave her alone, because when she gets an attack nobody helps her, so I have to be near her almost all the time.*
**Interviewer**	*How do you feel when separated from your person?*
**Respondent**	*Yea...... I don't feel happy because when I leave her alone and I go for a burial I don't be attentive the other side, my minds be behind thinking about her.*
**Interviewer**	*How does caring for a person with epilepsy influence your behavior?*
**Respondent**	*Her illness has not changed my behavior in any way..*

Case 27

SECTION B: PSYCHOLOGICAL BURDEN

Interviewer	How does caring for your sister influence the way you think about life?
Respondent:	On my side I am worried because when the sickness comes it attacks her and she suffers a lot. Treating her becomes hard at times because it is hard for me to get money.
**Interviewer**	How does caring for a person with epilepsy interfere with your personal relationship with others?
**Respondent**	*People around us in our village fear to come near her because they think that when they come near her this sickness can easily be transmitted to them.*
**Interviewer**	*Do you have time for yourself, can you elaborate?*
**Respondent**	*I don't have enough time for myself because I fear sometimes to leave her alone, so I have to be near her most of the time, I used to have a shop but due to her sickness I had to close it up.*
**Interviewer**	*How do you feel when separated from your person?*
**Respondent**	*I don't feel happy because when I leave her alone and I go somewhere I don't settle the other side, my minds be behind thinking about her.*
**Interviewer**	*How does caring for a person with epilepsy influence your behavior?*
**Respondent**	*Her illness has not changed my behavior in any way..*

Case 30

SECTION B: PSYCHOLOGICAL BURDEN

**Interviewer**	How does caring for your person afflicted with epilepsy influence the way you think about life?
**Respondent:**	I am confused because I have tried everything to see to it that my child gets well all in vain sometimes I wish God could take her and she rests.
**Interviewer**	How does caring for a person with epilepsy interfere with your personal relationship with others?
**Respondent**	*I relate well with others caring for my child does not interfere with my relationship with people.*
**Interviewer**	*Do you have time for yourself, can you elaborate?*
**Respondent**	*The time I have is enough only when that attack come s that is my other work is affected and I remain cursing.*
**Interviewer**	*How do you feel when separated from your person?*
**Respondent**	*I feel bad, because I don`t know what may happen, to her that scares me the more and I don't delay from where I go.*
**Interviewer**	*How does caring for a person with epilepsy influence your behavior?*
**Respondent**	*Since i understand her condition and I know that one calls for a disease my behaviors have never changed in fact I treat her with a lot of care as a delicate person.*
