# Supplementary material for: Lived experiences of caregivers of persons with epilepsy attending an epilepsy clinic at a tertiary hospital, eastern Uganda: A phenomenological approach
Source: PLoS One. 2023 Jul 18;18(7):e0274373. doi: 10.1371/journal.pone.0274373 (PMC10353802; doi:10.1371/journal.pone.0274373)
Supplement: S1 Data — (ZIP) [file pone.0274373.s001.zip › Key Informants-healthworkers.pdf]

KI 1.

Interviewer	What is your perception of the way care givers feel while taking care of their sick persons?
Respondent	They think that for us we know, we can give good advise and treatment. Others think that it is witchcraft; they think that this disease is infectious.
Interviewer	How does your institutional programme deal with the stress related effects of the care givers?
Respondent	It employs health workers to assist the patients, there is also mental health Uganda there those patients who were once epileptic give testimonies they sensitize patients about compliance with treatment because this is the only way to control fits.
Interviewer	What is your role as a health worker in caring for the care givers?
Respondent	My role is to give health education, counsel them, I encourage them to continue with treatment for life because when some patients come they think that this disease is like malaria where they can take medicine for one month and stop, I also give nursing care to them.
Interviewer	What resources are available in taking care of patients and care givers here in Mbale Regional Hospital?
Respondent	We have epileptic drugs, patients provide meals for themselves but if there is one who doesn`t have a relative we have JENGA(a Christian based Non-Government Organisation) that helps needy patients to buy drugs, they provide food and at times they give the patients clothes. We also have social workers who take care of patients who come without their relatives.
Interviewer	How do you think care givers family/social relationships are affected because of caring for persons afflicted with epilepsy?
Respondent	It can be affected due to stigma other people say that this thing is contagious they even fear to share if it is a child the neighbours fear and stop their children from playing together with him or her. In my village I used to observe people serve this patients food on banana leaves they would refuse to use their plates thinking that the thing is contagious, this epileptic patients at times get irritable so their relatives can be disgusted them they can allege stories that everyone hates them, if it is a man he can chase away the wife, this change of behavior makes their care giver to get tired of them.
Interviewer	What symptoms do you think care givers of persons afflicted with epilepsy have?
Respondent	They can get tired very first because of looking after them, they can be stressed up, some worry that their patient is not going to recover because there are those patients who can fit continuously the probability of saving their lives is a half some of them end up dying because of these continuous fits this makes the care givers lose hope.
Interviewer	How do you think care givers personal well being is affected by their role?
Respondent	Most of them end up becoming poor because they are confused, people deceive them that traditional healers can solve their problem so they spend a lot of money and by the time they come to the hospital the patients condition may have deteriorated and I may not afford to buy the drugs that are needed to manage this severe form of epilepsy because they are very expensive.

Interviewer	What is your perception of the way care givers feel while taking care of their sick persons?
Respondent	They are always stigmatized, they fear that their patient is not going to be well again, others believe that their patient has been bewitched or cursed by either the clan or the people around them don`t wish them peace.
Interviewer	How does your institutional programme deal with the stress related effects of the care givers?
Respondent	We have the psychologists who normally have sessions with them and they teach them, they counsel them and they also get testimonies from one another.
Interviewer	What is your role as a health worker in caring for the care givers?
Respondent	My role is to teach them how to manage this patients ,I tell them about the danger areas that they should protect their patient from like the patient should be protected from fire places, water bodies or broken bottles because all these things can cause harm to the patient.
Interviewer	What resources are available in taking care of patients and care givers here in Mbale Regional Hospital?
Respondent	We have trained health workers, drugs are available, we also have beds for patients to sleep on in case they are admitted, however this beds are not protected on the sides so the patient can easily fall down when they are fitting.
Interviewer	How do you think care givers family/social relationships are affected because of caring for persons afflicted with epilepsy?
Respondent	They are mostly affected by stigma because people in the community they are not educated, they do not know much about this disease and how it presents they keep on attributing this to other things like witchcraft, curses and this disturbs the patients relative. Other patients never improve very well such patients entirely depend on their relatives they end up being a big burden because one has to take care of that person like a baby also the men and women who have this disease fear to marry or get married.
Interviewer	What symptoms do you think care givers of persons afflicted with epilepsy have?
Respondent	They show aggression some of them become so tough as a result of fatigue, others get stressed up because of the patient's condition.
Interviewer	How do you think care givers personal well-being is affected by their role?
Respondent	Because of stress they are ever thinking about how the patient will be at the end. They end up being lonely, psychologically tortured and live in segregation.

Interviewer	What is your perception of the way care givers feel while taking care of their sick persons?
Respondent	It depends on how people view sickness but on the whole they feel bad.
Interviewer	How does your institutional programme deal with the stress related effects of the care givers?
Respondent	We counsel them, we health educate them on the causes, we tell them that the disease is not infectious and we encourage them to take care of the patients properly by ensuring that they take their drugs as recommended, bring them back for review, feed them, avoid precipitating factors.
Interviewer	What is your role as a health worker in caring for the care givers?
Respondent	I encourage them to take good care of the patients, I also give health education and do counseling to the patients.
Interviewer	What resources are available in taking care of patients and care givers here in Mbale Regional Hospital?
Respondent	We have specialized personnel, their drugs are available and the mental health unit is fully functional it provides services to the patients 24 hours in a day.
Q	How do you think care givers family/social relationships are affected because of caring for persons afflicted with epilepsy?
Respondent	Resources are wasted, money is wasted for caring for them, time is wasted, and they are stigmatized and think that the disease is infectious.
Interviewer	What symptoms do you think care givers of persons afflicted with epilepsy have?
Respondent	They can develop depression and they are isolated in the communities.
Interviewer	How do you think care givers personal well-being is affected by their role?
Respondent	They may not eat well, they may not dress well, they don`t sleep well their general outlook is not good because they are drained of the resources.

Interviewer	What is your perception of the way care givers feel while taking care of their sick persons?
Respondent	One of the positive way is that they feel they need to offer help to enable their relatives to get better. In case they observe behaviours they come for treatment. On the other side some of the care givers have almost given up their businesses the reason is that this is a chronic condition which requires resources, some of them feel that they have spent too much money on treating the patient but they don't get cure so they are withdrawing their help.
Interviewer	How does your institutional programme deal with the stress related effects of the care givers?
Respondent	We keep on encouraging the care givers not to give up, to always bring them for review, we give them information on how the sickness progresses so that they understand that this is a chronic problem and the patients must always be attended to and brought for reviews.
Interviewer	What is your role as a health worker in caring for the care givers?
Respondent	`my role to the care givers is to give them information, I protect them because they are coming to a mental unit where some patients become aggressive and can harm them, counsel them about the sickness because they may be giving up on the care giving role so we encourage them not to give up
Interviewer	What resources are available in taking care of patients and care givers here in Mbale Regional Hospital?
Respondent	To the patient the health workers make a proper diagnosis, they provide drugs to them and they also offer professional care to the patients who are admitted. For the care givers we protect them by providing security in our premises.
Interviewer	How do you think care givers family/social relationships are affected because of caring for persons afflicted with epilepsy?
Respondent	Stigma affects the care givers family and the rest of the community. Sometimes the relationship between the families of the care givers and the community members is strained. At times some of these patients can become aggressive and get involved in fights with their family members. This affects the entire family.
Interviewer	What symptoms do you think care givers of persons afflicted with epilepsy have?
Respondent	They become depressed because of the challenges these patients go through some of them become anxious and complain of burnout.
Interviewer	How do you think care givers personal well being is affected by their role?
Respondent	Their social connection with others are cut down since most of them are close to the patients all the time their network with other friends is completely cut off. Some of them become ineffective at work, their productivity is lessened this can lead to suspension, dismissal or termination from work because they absentee from their work places most of the time

Interviewer	What is your perception of the way care givers feel while taking care of their sick persons?
Respondent	I think they are stressed, they are also stigmatized because people have many beliefs about epilepsy in the community some think that the patient got the disease because the family sinned or is cursed . this things lead to stress or stigma in the lives and families of the care givers.
Interviewer	How does your institutional programme deal with the stress related effects of the care givers?
Respondent	We have outreach programme whereby we go out to people with epilepsy in the community and talk to them about how they should take the medicine we tell them that epilepsy is a chronic illness and we encourage the care giver to support their persons who are afflicted with epilepsy. There also programmes on the radio where health workers have specific days to go and talk about conditions like epilepsy, they tell people about the services that they are offered at the hospital and discourage them from seeking help from the traditional healers. Still when they come to the hospital, we give continuous health education to them.
Interviewer	What is your role as a health worker in caring for the care givers?
Respondent	My role as a health worker is to give the care giver enough information about this condition, I offer counseling to them, I also identify the care givers who are stressed and refer them to the psychologist who attends to them.
Interviewer	What resources are available in taking care of patients and care givers here in Mbale Regional Hospital?
Respondent	Drugs are always available; we have trained health workers who are specialized and give in enough time and special medical care to the patients and their care givers.
Interviewer	How do you think care givers family/social relationships are affected because of caring for persons afflicted with epilepsy?
Respondent	I thank families can get affected, for example if the woman is the one in the hospital and the man remains at home to take care of the children for a long time, he may think of marrying another woman. Most patients are still isolated in community they do not eat together with other people because they believe and fear that they can be infected when they share the same plates or cups with this patient.
Interviewer	What symptoms do you think care givers of persons afflicted with epilepsy have?
Respondent	Caring for this patients causes stress to the care givers, some report that they don't sleep well at night because of the thoughts and worries that they get, some of them believe that consuming alcohol can help them to forget about their problems so they end up abusing alcohol
Interviewer	How do you think care givers personal well-being is affected by their role?
Respondent	The care givers get stressed up, their modes change, they become sad and lose interest in the activities they used to enjoy.

Interviewer	What is your perception of the way care givers feel while taking care of their sick persons?
Respondent	Some people feel it as a burden because they have to take care of the patient, when they are away from home they are worried and wonder if the patient is safe because the attacks comes at any time this confuses them whereas some people feel it is a burden, other people go ahead and find someone to take care of their relatives as they go for their duties.
Interviewer	How does your institutional programme deal with the stress related effects of the care givers?
Respondent	We have the JENGA programme which holds care giver sessions very Wednesday where the care givers share the same experiences on how they handle their patients, how they feel when the patients are under attacks and that helps clear some misconceptions and helps them understand they are not alone in the struggle these sessions are attended by nurses, social workers, psychologist among others who answer questions from the care givers.
Interviewer	What is your role as a health worker in caring for the care givers?
Respondent	My role as a health worker is to help this care taker to appreciate that epilepsy is a life long illness and it is just like any other disease so taking care of a patient with this illness is just like taking care of a patient with any other illness. They have to appreciate that these fits can be controlled though it takes long and someone has to be on drugs throughout their life.
Interviewer	What resources are available in taking care of patients and care givers here in Mbale Regional Hospital?
Respondent	We have drugs and trained health workers who attend to the patients and their care givers.
Interviewer	How do you think care givers family/social relationships are affected because of caring for persons afflicted with epilepsy?
Respondent	Where I see it affects the relationships is that the family is a stigmatized by the neighbours due to the misconception by the community. Some families are segregating people say that they have demons there are given lots of names even other people get discouraged from marrying such families.
Interviewer	What symptoms do you think care givers of persons afflicted with epilepsy have?
Respondent	Sometimes they get fatigued because when the patients have a fit they can bite them when they are unconscious, they can also beat them because they become so energetic and in the process of restraining them they become tired.
Interviewer	How do you think care givers personal well-being is affected by their role?
Respondent	They have to deprive themselves of something's to make sure that they buy medicine for their patients especially when these drugs lack from the hospital. Most of these drugs are expensive so you find that sometimes they have use the limited resources that they have in taking care of the patient.

Interviewer	What is your perception of the way care givers feel while taking care of their sick persons?
Respondent	For them what they feel is that they have to be there for the client to ensure that they get the right drugs, at the right time, in the right dose. They also give a report about the patient's progress to the health workers.
Interviewer	How does your institutional programme deal with the stress related effects of the care givers?
Respondent	When the care givers come adequate information is passed to them on how to manage the patient's condition and their misconceptions about this disease is cleared.
Interviewer	What is your role as a health worker in caring for the care givers?
Respondent	As a counselor I talk to the patient's and help them come up with solutions to the challenges that they face. I also encourage them not to give up on caring for their sick relatives.
Interviewer	What resources are available in taking care of patients and care givers here in Mbale Regional Hospital?
Respondent	We have an adequate number of staff, the drugs are available and we pass on information about epilepsy to the patients and their care givers by conducting health education talks.
Interviewer	How do you think care givers family/social relationships are affected because of caring for persons afflicted with epilepsy?
Respondent	The care givers who work some times are forced to stop working so that they can attend to their beloved ones, this affects their families in the sense that they may not be able to provide them with the basic needs. The care givers are also restricted from moving because this clients can get an attack if they are left alone without any one to take care of them so they are forced to stay in one place to monitor the patient and in so doing they end up losing their social network .
Interviewer	What symptoms do you think care givers of persons afflicted with epilepsy have?
Respondent	They may get fatigued because sometimes they don't get enough rest at night especially the patients get an attack.
Interviewer	How do you think care givers personal well being is affected by their role?
Respondent	They are stigmatized and discriminated in society some care givers are abandoned by their family members for stance the husband can abandoned the wife because he might think that the cause of the disease is from the wife's side.

Interviewer	What is your perception of the way care givers feel while taking care of their sick persons?
Respondent	The way I look at the care givers they feel burden because they are dealing with a chronic disease and the patient can not do much for themselves. It is not easy for the care takers.
Interviewer	How does your institutional programme deal with the stress related effects of the care givers?
Respondent	We have radio talks whereby we go to the radio stations and present about epilepsy of course people listen in the community and they sensitized then we answer some questions which they ask within the department when they come for their refill for drugs we give them continuous health education talks on their specific clinic days, on the wards we monitor them until when the fits stops and also we educate the care givers.
Interviewer	What is your role as a health worker in caring for the care givers?
Respondent	I give them knowledge about the disease, I answer the questions that they ask and give counseling to them as well.
Interviewer	What resources are available in taking care of patients and care givers here in Mbale Regional Hospital?
Respondent	We have drugs, we have security, we have beds and mattresses the patients use when they are admitted. There is an NGO (JENGA) which is responsible for providing food, drugs, clothes and transport to the needy patients who come to the hospital without their care givers.
Interviewer	How do you think care givers family/social relationships are affected because of caring for persons afflicted with epilepsy?
Respondent	They are affected when they see the patient is not improving; there are some beliefs that when your person sits with that one with disease, he or she is also attacked. It is also difficult for a girl child or male child with this disease to marry or get married because of the stigma that is associated with this disease. Families of patients suffering from epilepsy feel hated.
Interviewer	What symptoms do you think care givers of persons afflicted with epilepsy have?
Respondent	They are stressed and because of over thinking about the situation they can become depressed.
Interviewer	How do you think care givers personal well being is affected by their role?
Respondent	Its costly each time you have to travel to the hospital, you have to buy drugs, feed the patients and handle your own issues, poverty can set in.

Interviewer	What is your perception of the way care givers feel while taking care of their sick persons?
Respondent	Some of them expect to get cured with time, others believe that this disease is caused by witch craft and it is contagious.
Interviewer	How does your institutional programme deal with the stress related effects of the care givers?
Respondent	Peer support workers have health talks over the radio or on clinic days together with the psychologists. Care givers are taught how to manage this condition, they are told to protect the patient from dangerous places, to ensure that the patient complies with the treatment and is brought back for review.
Interviewer	What is your role as a health worker in caring for the care givers?
Respondent	I give them talk therapy this instills positive thinking in the care givers and also train others who have patients that are newly diagnosed so that they can cope with ease.
Interviewer	What resources are available in taking care of patients and care givers here in Mbale Regional Hospital?
Respondent	We have the personnel, who identify the patients, they treat them, and follow them up in the communities.
Interviewer	How do you think care givers family/social relationships are affected because of caring for persons afflicted with epilepsy?
Respondent	I think if there are no drugs at the hospital when their patient gets an attack they go to the witch doctors, also due to the lack of knowledge and ignorance in our communities most patients are stigmatized and isolated.
Interviewer	What symptoms do you think care givers of persons afflicted with epilepsy have?
Respondent	I think they get stressed up, for the sake of families with such children on the husbands blame their wives for producing a child with this condition.
Interviewer	How do you think care givers personal well being is affected by their role?
Respondent	In public places care givers of patients suffering from epilepsy feel ashamed and embarrassed if their relatives get an attack, such families hide these children from visitors and at work care givers children with this condition are less productive because they spend most of the time looking after the patient.

Interviewer	What is your perception of the way care givers feel while taking care of their sick persons?
Respondent	They believe that health workers are knowledgeable and the drugs that they give them can cure this disease.
Interviewer	How does your institutional programme deal with the stress related effects of the care givers?
Respondent	We normally have counseling sessions with the care givers and we also link up those who are helpless to the social workers for support.
Interviewer	What is your role as a health worker in caring for the care givers?
Respondent	I conduct health education talks, I offer counseling services, also appreciate the work that they do and I encourage them to continue taking care of the patient.
Interviewer	What resources are available in taking care of patients and care givers here in Mbale Regional Hospital?
Respondent	We have enough drugs stocked in place, we have trained, health workers, and we also get some support from Non- Government Organizations.
Interviewer	How do you think care givers family/social relationships are affected because of caring for persons afflicted with epilepsy?
Respondent	Financially they are strained because they have to transport themselves from the hospital, they have to buy drugs for the patient and cater for the entire family as well.
Interviewer	What symptoms do you think care givers of persons afflicted with epilepsy have?
Respondent	I think they can develop headache because of thinking so much about the patient's condition that has no cure. Also they can have disrupted sleep patterns because epilepsy attack come even at night and interfere with their sleep.
Interviewer	How do you think care givers personal well being is affected by their role?
Respondent	There social network is affected because they always have to keep at home and take care of the patient. Some of them end up resigning at work in order to take care of their beloved ones and others become financially drained because its expensive to maintain patient with a chronic illness.
