# Supplementary material for: Lived experiences of caregivers of persons with epilepsy attending an epilepsy clinic at a tertiary hospital, eastern Uganda: A phenomenological approach
Source: PLoS One. 2023 Jul 18;18(7):e0274373. doi: 10.1371/journal.pone.0274373 (PMC10353802; doi:10.1371/journal.pone.0274373)
Supplement: Attachment — Submitted filename: Review Comments.pdf [file pone.0274373.s003.pdf]

Review Comments

This paper highlights the lived experiences of care givers of persons with epilepsy attending the epilepsy clinic at Mbale regional referral hospital of eastern Uganda. I am glad to have reviewed it.

Overall: This is an engaging paper with a well written abstract, a clear aim, well described methodology and well-presented findings that suggest that caregivers of persons with epilepsy various burdens, including psychological, social, economic, and physical burdens.

Abstract and Introduction: The authors have provided a well written abstract, summarising the research question and the key findings. The authors took time to identify existing literature on the topic and justified how their study relates to the available literature. However, these sections could benefit from proofing of the original word document to address any grammatical and typographical errors.

Methodology: The methods are described in sufficient details, making the study technically sound although I am not an expert on the method of data analysis used for this particular study. This section could benefit from proofing of the original word document for rephrasing of some of the sentences (for example, sentence 166-168) to address grammatical and typographical errors.

Results, Discussions and Conclusion

The results are well presented in the various thematic areas that address the aim of the study and support the conclusion of the study. However, the study fails to discuss the limitations of the approach used to address the aim of the study and the implications of the findings of the study in the discussion. Thus, the authors should revisit the discussion to acknowledge the possible strengths and limitations of the study (Researcher or Interviewer induced bias, Validity Vs Reliability, Language Barrier, Highly qualitative nature of findings), highlight how the limitations may have been addressed and include the implications for practice of the findings of the present study to Uganda and Global Community. In addition, the authors should consider proofing all the sections of the paper to address grammatical and typographical errors to improve the flow and readability of the text. For instance, sentence 569-579 appears to be incomplete and this means the conclusion may require rephrasing.

Recommendations

While the recommendation for continuity of current activities being done by the mental health practitioners for patients with epilepsy and their care givers is a good idea, it would be good to make recommendations of solutions that address policy initiatives around the different themes of burdens experienced by the care givers and make recommendations for future research that may provide additional data on the burden being experienced by caregivers for patients with epilepsy. For instance, the authors could consider making suggestions of Longitudinal studies that can study the burden experienced by caregivers of patients with epilepsy, associated factors and offers solutions or consider the determinants of caregiver burden and its impact on quality of life (QOL) for caregivers.

Thank you for providing this piece of research. I am certain that with the above suggested revisions, it will be a great paper and worth the read.
