# Supplementary material for: Lived experiences of caregivers of persons with epilepsy attending an epilepsy clinic at a tertiary hospital, eastern Uganda: A phenomenological approach
Source: PLoS One. 2023 Jul 18;18(7):e0274373. doi: 10.1371/journal.pone.0274373 (PMC10353802; doi:10.1371/journal.pone.0274373)
Supplement: Attachment — Submitted filename: Reviewer notes.docx [file pone.0274373.s002.docx]

Thank you for the opportunity to review this manuscript focused on understudied lived experiences of caregivers of patients with epilepsy in Uganda. Findings will improve care of both the patients and supports for family caregivers.

Here are suggestions for strengthening the manuscript:

Lines 67-68. review and revise sentence structure. missing a word or comma?

Line 74. stay consistent with either caregivers or care giver.

Line 83-84. the numbers are a bit confusing! where did the remaining (831-295) patients go? were they admitted to inpatient?

Line 87-88. need reference to support this! or is your conclusion about insufficient knowledge of caregiver experiences solely in this one clinic?

Lines 111-112: Introduction of health workers was a surprise. Consider introducing this earlier say in purpose statement. You could say that the caregivers lived experiences will be elicited directly from them and their health workers who work with them in care of the patients. Alternatively give an explanation or the why for their inclusion somewhere in lines 109-120.

Line 123. no reference was provided in support of sample size and saturation info.

Line 132-133. this is a surprise! give more context and detail, such as rationale etc.

Line 151. review and revise this sentence. maybe missing some words.

Line 166-167. review and correct sentence.

Line 244-247. the quote does not seem to fit the sub-theme description preceding it.

Line 325-330. this section was a bit hard to follow.

Line 362. incorrect use of ; need to review entire document for greater grammar.

Line 540. use person-centered or strength-based language, like the patient or person with epilepsy instead of epileptic.

Line 551. Correct heads to headache.
