# Supplementary material for: Lived experiences of caregivers of persons with epilepsy attending an epilepsy clinic at a tertiary hospital, eastern Uganda: A phenomenological approach
Source: PLoS One. 2023 Jul 18;18(7):e0274373. doi: 10.1371/journal.pone.0274373 (PMC10353802; doi:10.1371/journal.pone.0274373)
Supplement: Attachment — Submitted filename: Response to Reviewers.docx [file pone.0274373.s004.docx]

Samuel Olowo,

Busitema University Faculty of Health sciences,

P.O Box 1460, Mbale, Uganda.

The Editor PLOS ONE

Dear Editor,

We thank you and the reviewers for the generous comments on our manuscript entitled **‘‘Lived experiences of caregivers of persons with epilepsy attending an epilepsy clinic at a tertiary hospital, eastern Uganda: A phenomenological approach ’’**.

We have editied the manuscript to address the concerns raised.

In particular we have summarized the comments as highlighted by reviewers and editors and their corresponding responses from the authors in a table format accompanying this letter.

We believe that the manuscript is now suitable for publication in PLOS ONE Journal

Samuel Olowo

Busitema University Faculty of health sciences

P.O Box 1460, Mbale, Uganda.

On behalf of all authors

| SNo. | **Academic editor/Reviewer (s) points raised** | **Authors’ response** |
| --- | --- | --- |
|  | **Journal Requirements:** |  |
|  | Please review your reference list to ensure that it is complete and correct. | Thank you very much for this guiding comment:  The entire reference list was reviewed to ensure that it is complete and correct. |
|  | Any changes to the reference list should be mentioned in the rebuttal letter that accompanies your revised manuscript. | Thank you very much for this guidance:  The changes made in the reference list have been mentioned here. |
|  | If you have cited papers that have been retracted, please include the rationale for doing so in the manuscript text, or remove these references and replace them with relevant current references. If you need to cite a retracted article, indicate the article’s retracted status in the References list and also include a citation and full reference for the retraction notice. | No retracted papers have been cited in the entire document. Howeever, some references especially those that currently can’t be downloaded or those where only the citation is available online and not the readable articles have been deleted and replaced with suitable alternatives.  To be specific the following references were deleted and replaced with sutable alternative:  9. Karim N, Ali A, Deuri SP. Care burden in epilepsy: A study from northeast India. Int J Res Dev Health. 2014;2(3):84-9.  10. Senthil M, Kiran M. Family interaction pattern among caregivers of patients with epilepsy and alcohol dependence. IOSR Journal of humanities and social science (IOSR-JHSS). 2015;20(9):01-9.  38. Janak E. Bracketing and bridling: Using narrative reflexivity to confront researcher bias and the impact of social identity in a historical study. Philanthropy & Education. 2018;1(2):82-93.  12. Jabor HA, Mohammed SH. Caregivers Knowledge about Psychosocial Problems of Epileptic Patients in AL-Diwanyia Governorate. Indian Journal of Forensic Medicine & Toxicology. 2019;13(2).  **The reference below was revised in terms of contents like the ‘DOI’:**  11. Anjum W. SCHIZOPHRENIA AND EPILEPSY. The Professional Medical Journal. 2017;24(05):757-66. |
|  | **Additional Editor Comments:** |  |
| 1. | The ethical considerations section is too wordy with lots of repetitions, lines 192-194 similar with 199-201; 199-203 with 204-206. Please avoid repetitions. | Thank you for this guiding comment:  The ethical considerations section was revised so that it’s not too wordy with no repetitions |
| 2. | Some of the quotes remain too long and may reduce readership, it would be better to summarize them in a table under the various themes so as to enhance easy reading. | Thank you very much for this guiding comment:  The long quotes in the entire document were shortened and others substituted with shorter alternatives from the original transcripts.  The quotes have been further summarized in a table under different themes to enhance easy reading. |
| 3. | Th discussion and limitations needs to be shortened and some sections deleted like lines 529-531, it would be nice to focus on the major study findings. | Thank you very much for this guiding comment:  The discussion and limitations were revised to make them shorter. Other Sections for example 529-531 were deleted. |
| 4. | Focus the limitations and make them concise and clear. | Thank you very much for this comment:  The limitations were revised to make them concise and clear |

********************************END*****************************************
