# Supplementary material for: Lived experiences of caregivers of persons with epilepsy attending an epilepsy clinic at a tertiary hospital, eastern Uganda: A phenomenological approach
Source: PLoS One. 2023 Jul 18;18(7):e0274373. doi: 10.1371/journal.pone.0274373 (PMC10353802; doi:10.1371/journal.pone.0274373)
Supplement: Attachment — Submitted filename: Response to Reviewers.docx [file pone.0274373.s005.docx]

| S/N | **Academic editor/Reviewer (s) points raised** | **Authors’ response** |
| --- | --- | --- |
|  | **Reviewer 1**.**:** |  |
| 1 | The authors need to back up their statements in the introduction, for example “the outpatient clinic recruits 295 patients in July” please reference | Thank you very much for this guiding comment:  The “295 patients” were obtained from a review of health management information system records. Furthermore, this information was not only for one month. The number of new cases was for the whole financial year (July 2016 to June 2017). |
| 2 | Eligibility criteria needs to be written well for both care takers and health care providers, | Thank you very much for this guiding comment:  The Eligibility criteria for both caretakers and healthcare providers were rewritten well |
| 3 | how were health workers selected? Was stratification done for the participants | Thank you very much for this guiding comment:  However, stratification was ensured while interviewing the participants to include psychiatric nurses of which they were six, two psychiatric clinical officers, one counsellor and one peer support. The aim was to ensure accurate and in-depth understanding of the lived experiences of the caregivers from the perspective of the different healthcare professionals involved in their care since management of epilepsy requires an interprofessional approach. |
| 4 | The authors need to discuss their results succinctly, the major findings first and then a fitting explanation, | We are grateful for this insightful comment:  The discussion of the results were revised with major findings first and later fitting explanation(s) followed |
| 5 | the conclusion and recommendations have to be re written, conclusions drawn from the findings and the implications too. | Thank you very much for the guiding comment:  The recommendations and conclusions were revised. The conclusions and implications of the study were derived from the study findings. |
|  | **Reviewer 2** |  |
| 1. | However, the paper needs notable reworking, and would strongly benefit from all of the authors reviewing it with an eye towards grammar and typical publication standards (some examples below). | Thank you for this observation:  Reworking of the paper was ensured. The entire document was revised in terms of grammatical and typographical errors to meet a publication standard |
| 2. | I would suggest that a senior member of the team review and guide the rework, and that additional support be added to the team if needed. | Thank you very much for this guiding suggestion:  All the senior members of the team reviewed and guided the reworking of the entire document |
| 3. | Open ended questions with follow-up probes are typically considered a structured interview guide-- depends on order and flexibility with the questions. | Thank you for this highlight:  This was revised to make it clearer and concise |
| 4. | Examples related to grammar and problems with referencing: | Thank you very much for this observation:  All the grammatical errors were revised in the entire document |
| 5. | -in the abstract "Feelings" is midsentence and capitalized. | Thank you very much for this guiding comment:  Feelings has been rewritten and uncapitalized. |
| 6. | -The introduction states that the ILAE recognizes epilepsy as an NCD, which is true. Epilepsy is recognized as a chronic major NCD by ILAE, the World Health Organization, etc. But the reference attached to this (3) is not an ILAE publication. It would be best to reference a publication of the ILAE (or better, the WHO), and then reference the Singh paper for the worldwide statistic (best done by having these 2-3 references together at the end of the sentence). | Thank you very much for this insightful comment:  The whole statement was reframed with WHO statistics |
| 7. | The incidence of epilepsy has been reported to be high in Uganda with over 156 new cases of epilepsy per 100,000 people each year and an increasing prevalence of 13.3% [7] -- this paper reports the crude prevalence at 13.3, what paper holds the 156/100,000 rate? Please reference that primary source. | Thank you very much for the guiding comment:  The primary source for 156/100,000 incidence rate has been referenced |
| 8. | Line 80 has 13 referenced twice | Thank you very much for this guidance:  The referencing of line 80 was revised |
| 9. | -While epilepsy is a common phenomenal in Uganda: phenomenon | Thank you for this guidance:  The word “phenomenal” was revised to mean the actual intended word “phenomenon” |
| 10 | -Lines 88-94 are given as justification for the study. The number of people attending the clinic, their gender, etc., should not drive a research question. Instead, lack of knowledge of caregiver's lived experience (regardless of number) drives the question. | Thank you very much for this insightful comment:  The statement was revised to make “lack of knowledge of caregivers’ lived experiences” as the justification for the study |
| 11 | -bed occupancy averagely 359 | Thank you for this guiding observation:  This sentence was revised to make it clear. |
| 12 | -147: the interviews guide was | Thank you for this comment:  This was revised to “interview guide” |
| 13 | -150: like leading question | Thank you very much for this guiding comment:  “Leading question” was revised to mean “leading questions” |
| 14 | 183: "till" is not standard English | Thank you very much for this guiding comment:  ” till” was revised to a standard English “until” |
|  | 186: Conformability of the study is demonstrated by how the empirical the empirical data is in line with the study findings. | Thank you for this observation:  This was revised to “Conformability of the study is demonstrated by how the study findings align with the findings of other related studies”. |
| 15 | The Ethical considerations section is far too long. Please review recent publications for examples of typical reporting. | We are grateful for this insightful comment:  The Ethical considerations section was revised to make it short clear and concise. |
|  | **VERY IMPORTANT:** |  |
| 16 | Tables 1, 2, and 3, 4 have theme/subthemes listed and selected illumative quotes. Then the text has more quotes. This presents the data in a split fashion, when it is necessary to pick one. The tables are efficient, and thus the text should be limited to introducing the content of the table, not providing more content. | Thank you very much for this guiding comment:  The quotes have been majorly only summarised in the tables and the text was limited to introducing the content of the table. The table has been linked to the text through cross-referencing. |
| 17 | The Discussion needs to not only relate the current findings to existing papers, as it does, but also needs to professionally and succinctly offer what NEW information was learned from this study. | Thank you very much for this guiding observation;  The discussion was revised to include new information that was learned from the study in addition to the findings that exits in the published papers. |
| 18 | Can the sample be described by patient’s age, type/duration/severity of epilepsy? These factors could influence caregiver burden and should be reported. | Thank you very much for this observation.  The patient’s age, type/duration/severity of epilepsy was not considered while sampling. However, the information on duration of epilepsy was captured in the results section under demographic characteristics. The findings demonstrated that About 13(43%) of the participants reported that their patients had epilepsy for more than 5 years, 8(27%) fell in the range between three to five years, 8(27%) of the participants fell in the range between one to two years and 10(33%) of the participants reported that their participants had suffered from epilepsy for less than a year. |
| 19 | Recommendations should go in the Discussion unless this journal routinely breaks it into a separate section. | Thank you very much for this guidance.  According to the Journal guidelines, the recommendations break into a separate section from the discussion. |
| 20 | “The collection and analysis of the data were done simultaneously”. I do not think this is accurate. Thematic analysis requires transcription and careful study of results, which would occur well after the discussion that collected the data. | Thank you for this guiding comment:  This was revised to make it clearer |
| 21 | “_an inductive thematic analysis.... thorough reading and understanding of the transcripts ensured to obtain an overall sense...Line coding...A thorough review of the transcripts were ensured and later a list of codes....” This could be made more succinct and stated once. If in the methods the authors describe the thematic analysis approach well, it does not need to be stated that the content was read and understood, etc. This is where a review of the paper by the senior member of the team may be helpful to make reporting concise and clear. Regards, | Thank you very much for this guiding comment:  We have revised the data analysis section to make it concise and clear. |

********************************END**************************************************************
