# Supplementary material for: Lived experiences of caregivers of persons with epilepsy attending an epilepsy clinic at a tertiary hospital, eastern Uganda: A phenomenological approach
Source: PLoS One. 2023 Jul 18;18(7):e0274373. doi: 10.1371/journal.pone.0274373 (PMC10353802; doi:10.1371/journal.pone.0274373)
Supplement: Attachment — Submitted filename: reviewer comments.docx [file pone.0274373.s006.docx]

**20/6/2023**

**Introduction**

it’s too lengthy with a lot of unrelated redundancy, kindly remove line 55 to 63 and lines 70 to 77.

**Methods**

1.Rephrase/ rewrite the statement for eligibility age of consent.

2.How where caregivers selected, given the low educational status?

3.There is	a lot of repetition in this section, that needs to be shortened to remove the repetition.

4. How was stratification done to ensure full coerciveness of the group and participation?

**Results**

This is an erroneous statement “With regards to education, the majority, 15(50%) of the participants never acquired formal education”. Yet 15 0ut of the 40 participants, Rewrite

Line 233, start with “among the care givers” 10% were biological fathers……” instead of participants and7 % had no relationship

With regards to education 7(70%) of the participants had ap with the patients. “Start with majority”

**Discussion**

1. There is	a lot of redundancy	and repetition, “This study 470 discusses the lived experiences of caregivers into four interrelated themes. These include psychological, social, physical, and economic burdens. The caregivers perceived epilepsy as a major burden interfering with different aspects of their lives” this can be removed.

2.The	authors	in this paragraph state that “Different psychological burdens surfaced among caregivers. Worries about day-to-day living 474 and future life of their persons were expressed by the participants. Their explanation is shallow and needs more meat to make a better read. They should also expound on	prognosis of the patient which is clearly expressed in the subtheme.

3.Page 24, regarding	sleep, this was reported by what was felt/experienced by the	caregivers, however the authors conclude how this affects SUDEP in patients with epilepsy yet not they never assessed patients with epilepsy. This is a a strong assumption. The authors need to discuss their results

4.Throughout	the read the effect of stigmatization on both the caregivers and	people living with	epilepsy needs to be well discussed as it’s a critical issue which came up in quotes

5. Among the study limitations, was the sampling method “snowball” kindly include and	discuss

**Implications and conclusions**

1.The implications have barely touched on the study leaving out the important messages, and still the conclusion. The authors need to draw	conclusions	from the study findings, they miss some major conclusions which would enrich and enlighten the study
